# Structural and Functional Insights into WRKY3 and WRKY4 Transcription Factors to Unravel the WRKY–DNA (W-Box) Complex Interaction in Tomato (*Solanum lycopersicum* L.). A Computational Approach

**DOI:** 10.3389/fpls.2017.00819

**Published:** 2017-05-29

**Authors:** Mohd Aamir, Vinay K. Singh, Mukesh Meena, Ram S. Upadhyay, Vijai K. Gupta, Surendra Singh

**Affiliations:** ^1^Department of Botany, Centre for Advanced Study, Institute of Science, Banaras Hindu UniversityVaranasi, India; ^2^Centre for Bioinformatics, School of Biotechnology, Institute of Science, Banaras Hindu UniversityVaranasi, India; ^3^Department of Chemistry and Biotechnology, ERA Chair of Green Chemistry, Tallinn University of TechnologyTallinn, Estonia

**Keywords:** transcription factors, DNA binding domain, homology modeling, monophyletic origin, DNA-protein docking

## Abstract

The WRKY transcription factors (TFs), play crucial role in plant defense response against various abiotic and biotic stresses. The role of *WRKY3* and *WRKY4* genes in plant defense response against necrotrophic pathogens is well-reported. However, their functional annotation in tomato is largely unknown. In the present work, we have characterized the structural and functional attributes of the two identified tomato WRKY transcription factors, WRKY3 (SlWRKY3), and WRKY4 (SlWRKY4) using computational approaches. *Arabidopsis* WRKY3 (AtWRKY3: NP_178433) and WRKY4 (AtWRKY4: NP_172849) protein sequences were retrieved from TAIR database and protein BLAST was done for finding their sequential homologs in tomato. Sequence alignment, phylogenetic classification, and motif composition analysis revealed the remarkable sequential variation between, these two WRKYs. The tomato WRKY3 and WRKY4 clusters with *Solanum pennellii* showing the monophyletic origin and evolution from their wild homolog. The functional domain region responsible for sequence specific DNA-binding occupied in both proteins were modeled [using AtWRKY4 (PDB ID:1WJ2) and AtWRKY1 (PDBID:2AYD) as template protein structures] through homology modeling using Discovery Studio 3.0. The generated models were further evaluated for their accuracy and reliability based on qualitative and quantitative parameters. The modeled proteins were found to satisfy all the crucial energy parameters and showed acceptable Ramachandran statistics when compared to the experimentally resolved NMR solution structures and/or X-Ray diffracted crystal structures (templates). The superimposition of the functional WRKY domains from SlWRKY3 and SlWRKY4 revealed remarkable structural similarity. The sequence specific DNA binding for two WRKYs was explored through DNA-protein interaction using Hex Docking server. The interaction studies found that SlWRKY4 binds with the W-box DNA through WRKYGQK with Tyr^408^, Arg^409^, and Lys^419^ with the initial flanking sequences also get involved in binding. In contrast, the SlWRKY3 made interaction with RKYGQK along with the residues from zinc finger motifs. Protein-protein interactions studies were done using STRING version 10.0 to explore all the possible protein partners involved in associative functional interaction networks. The Gene ontology enrichment analysis revealed the functional dimension and characterized the identified WRKYs based on their functional annotation.

## Introduction

Plants throughout the course of their entire development encounter challenges from exposure to various abiotic and biotic stresses. High temperature or temperature changes from freezing to scorching, salinity stress, water stress (drought), nutrient deprivation, and variable light conditions affect the overall growth morphology and physiological processes. However, plants respond well to these environmental stresses by developing various intricate mechanisms that work at multiple levels. The most common mechanism involves the extensive reprogramming of their transcriptome in a highly dynamic and temporal manner and is achieved through a complex network of proteins working as transcriptional factors. Transcription factors (TFs) provides a class of genes, having critical role in stress tolerance mechanisms and participate in the transcriptional regulation of stress responsive genes in the plants (Mickelbart et al., 2015). These trans-acting sequence-specific DNA binding specifically recognize the *cis*-acting promoter elements that leads to the formation of transcriptional complexes which modulate the fine tuning of gene expression and therefore, regulates the expression of stress-inducible genes (Xu et al., 2006; Mickelbart et al., 2015). The phenotypic plasticity against various stresses is achieved through differential gene expression that directs and integrates the multitude of some synergistic or antagonistic signals, thus allows plants to respond well under such extreme conditions.

WRKY transcription factors include the most crucial and largest families of plant transcriptional regulators, having diverse functional roles such as in the development of resistance against various plant pathogens, mitigation of the abiotic stresses, senescence, nutrient deprivation, embryogenesis, and hormone-dependent developmental programming (Bakshi and Oelmüller, 2014). They regulate the multiple biological stresses both biotic and abiotic to provide an indigenous defense response against pathogen-induced challenges (Chen et al., 2013; Yamasaki et al., 2013; Banerjee and Roychoudhury, 2015) or to tackle with environment-induced changes which includes abiotic stresses such as wounding, drought, salinity, heat, cold, and osmotic pressure (Tripathi et al., 2014). The fine tuning of the defense network associated genes can occur due to the direct modulation of immediate downstream target genes which may be either repressed or de-repressed in association with other members of the WRKY family or other proteins in both feed forward and feedback regulatory loops. In addition, WRKYs role in various plant developmental as well as reproductive processes including plant senescence, formation of root hair and trichomes, regulation of seed coat color, seed size, male synthesis of carbohydrate, and other secondary metabolites and male gametogenesis is well-documented (Luo et al., 2005; Guan et al., 2014; Jiang et al., 2016).

**Figure 1:**
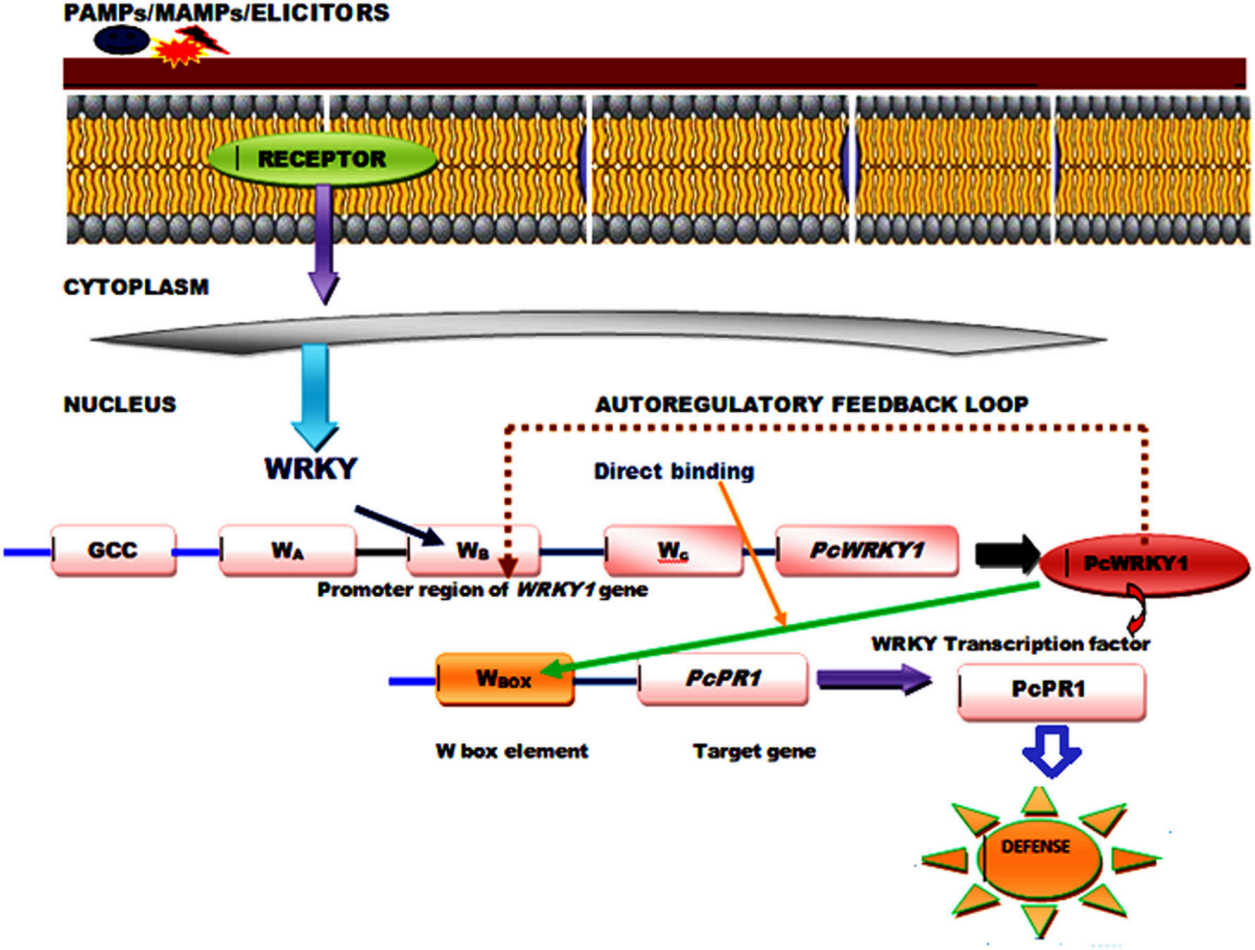
**A generalized model showing functional mechanism and role of PcWRKY1 (*Petroselinum crispum*) in PAMP induced WRKY gene regulation through direct binding and auto regulatory negative feedback loop**.

The WRKY proteins constitute a large transcription factor family widely distributed among plants (Agarwal et al., 2011) and recognized on the basis of a highly conserved WRKY domain that contains ~60 amino acids, comprised of highly conserved short heptapeptide WRKYGQK sequences at the N-terminus, and a C_2_H_2_ (C–X4–5–C–X22–23–H–X1–H) or C_2_HC (C–X7–C–X23–H–X1–C) zinc-binding motif at the C-terminus (Li et al., 2015). The RKYGQK residues of the core motif and the additional arginine and lysine residues of the WRKY domain are responsible for the interaction with the phosphate backbone of the seven consecutive DNA base pairs, including the GAC core (Yamasaki et al., 2013). However, the transcriptional binding of WRKY TFs is well-affected by the number of WRKY domains and different features of the zinc finger motifs that varies in between different members (Bakshi and Oelmüller, 2014). Based on the presence of number of WRKY domains and composition of their zinc finger motifs WRKY TFs can be divided into three groups (Groups I, II, and III) that can bind to the W-box DNA (C/T) TGAC(C/T) (Huang et al., 2012). Since, almost all the studied WRKY members recognize the TTGACC/T the W-box sequences the functional diversity observed in between the members and the specific regulation achieved by an individual protein is highly dependent on some additional mechanism other than DNA binding. It has been reported that WRKY transcription factors physically interact with a wide range of proteins playing significant roles in signaling, transcription and chromatin remodeling, and these interaction studies have provided necessary information regarding their action mechanism and mode of regulation. Moreover, a single WRKY gene may respond to multiple types of stresses, and then their proteins participate in various distinct processes as both positive and negative modulators. The complex functional mechanism of the signaling and transcriptional reprogramming by WRKY genes, following the stress conditions may involve the regulation mediated by protein-protein interaction, autoregulation and cross-regulation (Chen et al., 2012). The conserved motifs and slightly varied WRKY domain play crucial role in mediating complex functional protein-protein interaction observed between different WRKY members or those reported for WRKYs with other regulatory protein partners through both auto regulation or may involve cross regulatory mechanisms (Chi et al., 2013; Agarwal et al., 2014). The cross-regulatory pathway for their regulatory mechanism may involve several interacting proteins such as DNA binding proteins and other components including MAP kinases (Ishihama and Yoshioka, 2012), calmodulin (Park et al., 2005), VQ proteins (Chi et al., 2013), histone deacetylases (Glatt et al., 2011), E3 ubiquitin ligases (Miao and Zentgraf, 2010), and CC-NBS-LRR type R-proteins (Liu et al., 2016). The autoregulatory control mechanism could occur through negative feedback loop and direct binding of the pathogen associated molecular pattern (PAMP) induced WRKY genes and other expressed target genes. WRKY1 from parsley (*Petroselinum crispum*) has been reported to interact with the W-box promoter of its own gene as well as promoters of *PcWRKY3* and the marker gene *PcPR1*, as revealed through the chromatin immunoprecipitation (ChIP). PAMP-triggered early responses recruits PcWRKY1 to the three synergistically acting W boxes (W_ABC_), occupied constitutively by the prebound WRKY repressor molecules. Simultaneously, PcWRKY1 also binds to the W-box sequences present in the promoter of the target gene, *PcPR1* (Figure 1) that leads to the repression of *PcWRKY1* itself and activation of *PcPR1* (Turck et al., 2004). Moreover, a good example of positive feedback through auto regulation is provided by pathogen-inducible *WRKY33* gene in *Arabidopsis* whose expression is controlled by MAPK3/6 (Mao et al., 2011). ABA signaling regulators involve the role of WRKY18, WRKY40, and WRKY60 whose binding to W-box sequences lying in the promoter region of their target genes leads to overall repression of all the three WRKY genes (Yan et al., 2013).

During the past few years, many studies done on WRKY gene expression following the exposure of biotic stresses have demonstrated the role of WRKY3 and WRKY4 transcription factors in host defense mechanism. The pathogenic infection or incorporation of any pathogenic components or elicitor compounds that stimulate the expression of *WRKY3* and *WRKY4* genes to encode two structurally similar WRKY transcription factors WRKY3 and WRKY4 and are positive regulators in the plant resistance against necrotrophic pathogens (Lai et al., 2008). The role of tomato homologs for the *Arabidopsis* WRKY transcripts including *SlWRKY2, SlWRKY3, SlWRKY4, SlWRKY6, SlWRKY7, SlWRKY23, SlWRKY51, SlWRKY53*, and *SlWRKY71* have been demonstrated to be differentially upregulated following the infection caused by *Cladosporium fulvum* in the susceptible host tomato plants (van Esse et al., 2009). The expression analysis of drought induced genes in wild tomato line *Solanum habrochaites* following the artificially induced drought conditions revealed the downregulation of *SlWRKY4* genes in both sensitive and tolerant lines with relatively more downregulated in the tolerant lines (Gujjar et al., 2014). Moreover, the defense signaling involved during tomato-root not nematode (RKN) interactions involve differential upregulation of *SlWRKY3, SlWRKY23*, and *SlWRKY33* against root knot nematodes in both compatible and incompatible interactions which indicates their important role in RKN infections (Bhattarai et al., 2008). In rice, the defense response against the rice sheath blight pathogen, *Rhizoctonia solani* is regulated by *WRKY4* (*OsWRKY4*) (Wang H. et al., 2015). In cotton (*Gossypium hirsutum*), *WRKY3* (*GhWRKY3*) gene is involved in diverse stresses and the transcripts of *GhWRKY3* have been found enhanced after infection with *Rhizoctonia solani, Colletotrichum gossypii*, and *Fusarium oxysporum* f. sp. *vasinfectum* (Guo et al., 2011). The role of *Poncirus trifoliata* WRKY3 transcription factor (*PtrWRKY3*) in defense response against fungal pathogen *Phytophthora citrophthora* is well-known (Şahin-Çevik et al., 2014). Some other members such as *Arabidopsis* WRKY33 (*AtWRKY33*) have been reported as a positive regulator of resistance with the help of other WRKY proteins and some unidentified signaling molecules against necrotrophic fungi such as *Alternaria brassicicola* and *Botrytis cinere*a (Zheng et al., 2006; Birkenbihl et al., 2012). Moreover, the molecular complementation and gene silencing studies have demonstrated that tomato WRKY33 genes (*SlWRKY33A* and *SlWRKY33B*) perform the critical role similar to those of *AtWRKY33* (Zhou et al., 2015).

The genome-wide computational analysis revealed the presence of total 81 WRKY genes in tomato genome (Huang et al., 2012). However, the majority of WRKY genes in tomato are still uncharacterized or available with unknown biological functions. Till date, we do not have sufficient biological information about the functional attributes of tomato WRKY genes, their possible chromosomal localization, functional redundancy observed between different WRKYs and most important tomato WRKY transcription factors with overlapping functions (Huang et al., 2012). Furthermore, the comprehensive knowledge of the functional mechanism underlying the DNA binding, signaling cascades, conserved residues for making these interaction more feasible is critical and essential for effective gene regulation. The present work focussed on unraveling the structural and functional attributes of two important WRKYs (WRKY3 and WRKY4) in tomato. We have analyzed the DNA-protein interaction and protein-protein interaction using computational approaches to explore the functional residues involved in DNA binding and the probable protein interacting partners that got involved in WRKY protein signaling. The gene ontology enrichment analysis predicts the functional dimension of WRKY proteins on the behalf of their molecular function, biological processes in which they get involved and their possible cellular location.

## Materials and methods

### Sequence alignment and phylogenetic analysis

*Arabidopsis* WRKY3 (*AtWRKY3*) gene (Locus: AT2G03340) and WRKY4 (*AtWRKY4*) gene (Locus: AT1G13960) were retrieved from The Arabidopsis Information Resource (TAIR) database https://www.Arabidopsis.org/ (Lamesch et al., 2012) and the protein sequences for these Locus IDs WRKY3 (NP_178433.1) and WRKY4 (NP_172849.1) were retrieved from NCBI database. Further, NCBI BLAST server http://blast.ncbi.nlm.nih.gov/Blast.cgi (Altschul et al., 1997) was used for identifying the relevant sequential homologs available for these proteins in *Solanum lycopersicum*. The identified probable WRKY3 and WRKY4 proteins from *S. lycopersicum* (SlWRKY3 and SlWRKY4) were used for sequential classification and phylogenetic studies. The alignment results were checked by the BioEdit (Hall, 1999) tool. The UPGMA method was used to construct the phylogenetic tree and the tree was inferred by bootstrap phylogenetic inference using MEGA 6 suite http://www.megasoftware.net/ (Tamura et al., 2013) with 1,000 replicates. For inferring the homology and evolutionary relationship between all the identified members WRKY protein sequences were made for multiple sequence alignment using CLC bio workbench. The InteProScan http://www.ebi.ac.uk/Tools/pfa/iprscan/ (Jones et al., 2014) was used to obtain a “first-pass” profile of protein sequences potential functions. The functional WRKY domain region occupied in the SlWRKY3 and SlWRKY4 were identified using NCBI-CDD server http://www.ncbi.nlm.nih.gov/Structure/cdd/cdd.shtml (Marchler-Bauer et al., 2011) and ExPASy-Prosite scan http://prosite.expasy.org/scanprosite/ (de Castro et al., 2006). The distribution of potential motifs in both WRKY3 and WRKY4 proteins present across all the identified members were investigated using MEME Suite 4.1.1.2 (Multiple Expectation Maximization) for motif Elicitation http://meme.nbcr.net/ (Bailey et al., 2006). For the motif analysis, the selection of maximum numbers of motifs was set to 40 with an optimum motif width between 10 and 30 residues, with any number of repetitions. The Circos visualization tool was used http://circos.ca/ (Krzywinski et al., 2009) for the comparative analysis and the identification of similarities and differences for characterized WRKY proteins with the different members of tomato family and *Arabidopsis*. This was based on percentage similarity matrices obtained through phylogenetic clustering using Clustal W.

### Structural modeling

The identified protein sequences were taken for homology modeling and DNA protein docking analysis. For the structure modeling of the WRKY3 and WRKY4 domain from *S. lycopersicum* templates for homology modeling were searched using BLAST-P program of the protein data bank http://www.rcsb.org/pdb/ (Berman et al., 2000) with sequence similarities >90% to the available proteins in the PDB. The BLAST-P results revealed the most possible and closest templates available for modeling the WRKY domain structure for both SlWRKY3 and SlWRKY4 proteins. The three closest template structures that were further selected for protein modeling were C-terminal domain of AtWRKY4 (PDB ID:1WJ2), AtWRKY1(PDB ID: 2AYD) along with the complex of the C-terminal WRKY domain of AtWRKY4 and W-box DNA (PDB ID: 2LEX). The 3D structure prediction of SlWRKY3 and SlWRKY4 proteins was done using MODELLER module of Discovery studio 3.0 (accelrys.com; Shahi et al., 2013). The functional C-terminal domain (CTD) each from SlWRKY3 and SlWRKY4 were modeled and the modeled protein structures were made to superimpose over each others for finding the topological details using Superpose version 1.0 http://wishart.biology.ualberta.ca/SuperPose/ (Maiti et al., 2004). The backbone conformation of the predicted models (both SlWRKY3 and SlWRKY4) was further inspected based on the assessment measured in the form of backbone dihedral phi (φ) and psi (ψ) angles as depicted on the Ramachandran plot using PROCHECK module of the PDBsum server http://www.ebi.ac.uk/pdbsum/ (Laskowski et al., 2005) and further also confirmed with RAMPAGE http://mordred.bioc.cam.ac.uk/~rapper/rampage.php (Lovell et al., 2003). For the structural alignment, SALIGN-ModBase server https://modbase.compbio.ucsf.edu/salign/ (Braberg et al., 2012) was used The computationally predicted models were submitted in the protein modeling database (PMDB) https://bioinformatics.cineca.it/PMDB/ (Castrignano et al., 2006).

### DNA-protein interaction

The molecular interaction studies in between the W-box DNA and the protein sequences from SlWRKY3 and SlWRKY4 that constitutes the domain structure was done using Hex 8.0 molecular docking software (Macindoe et al., 2010). The parameters used for docking were correlation type: Shape+Electro+DARS; FFT Mode—3D fast lite; Grid Dimension—0.6; Receptor range—180; Ligand range—180; Twist range—360; Distance range—40. Further, the docked complexes were analyzed using visualization module of DS Studio 3.0 for their further interaction studies.

### Protein-protein interaction

The functional protein interactive associative network for SlWRKY3 and SlWRKY4 were searched using STRING (Search Tool for the Retrieval of Interacting Genes/Proteins database version 10.0) http://string-db.org/ (Szklarczyk et al., 2015). The active interaction sources were set based on the seven parameters including experiments, co-expression, gene fusion, co-occurrence, databases, textmining, neighborhood. The interactive scores were analyzed at all the confidence levels with interactions from both shell of interactors. The Predicted Tomato Interactome Resource (PTIR) http://bdg.hfut.edu.cn/ptir/index.html (Yue et al., 2016) was used to explore and validate all the possible functional interactive partners involved in WRKY signaling cascades.

### Structural and functional classification: CATH/gene3D server

The structural classification of the identified protein sequences was done using CATH server http://www.cathdb.info/ (Sillitoe et al., 2015). The functional classification of the identified CATH superfamilies were done using FunFHMMer http://www.cathdb.info/search/by_funfhmmer (Das et al., 2016) which scans the input protein sequences against CATH FunFam HMMs and the functional annotation were further analyzed based on gene ontological terms. The identified GO terms were fetched and a hypergeometric distribution test analysis was conducted using the REVIGO web server http://revigo.irb.hr/ (Supek et al., 2011). The identified GO terms were further confirmed for their probable subcellular localization and functional GO annotation using CELLO2GO web server http://cello.life.nctu.edu.tw/cello2go/ (Yu et al., 2014).

## Results

### Sequence alignment

**Figure 2:**
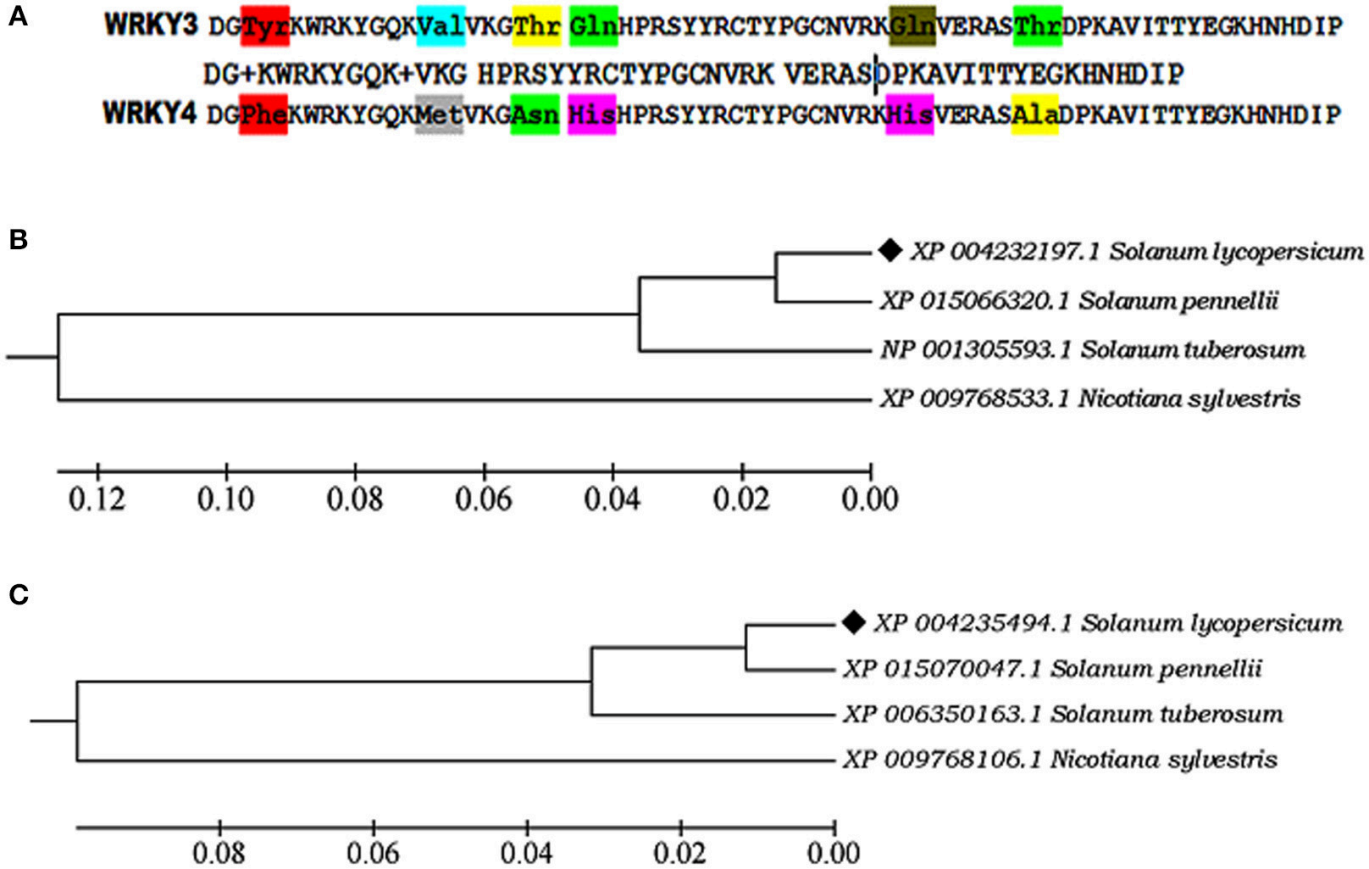
**(A)** Sequence alignment of the functional domain region from SlWRKY3 and SlWRKY4. The substituted amino acid residues have been shown in three letter codes **(B)** Phylogenetic tree showing the evolutionary origin of SlWRKY3 **(C)** Phylogenetic tree for evolutionary origin of SlWRKY4.

The sequence alignment showed AtWRKY3 and AtWRKY4 sequences were found to be most closely related to its tomato (*Solanum lycopersicum*) homologs WRKY3 (XP_004232197.1) and WRKY4 transcription factor (XP_004235494.1), respectively, based on the compositional matrix adjust methods with having identities 53/59 (90%) and positivity 55/59 (93%). Sequence alignment in between SlWRKY3 and SlWRKY4 revealed the maximum conservation of amino acid residues with some substitutions (Figure 2A).

### Database search and comparative phylogeny

Phylogenetic investigation for the evolutionary emergence of SlWRKY3 (XP_004232197.1) and SlWRKY4 (XP_004235494.1) revealed their monophyletic origin from their wild homologs *Solanum pennellii* (XP_015066320.1; Figure 2B) in case of SlWRKY3 and *S. pennellii* (XP_015070047.1; Figure 2C) for SlWRKY4. The multiple sequence alignment from WRKY3 protein sequences available for all the members showed the maximum conservation of amino acid residues across the divergent species in both WRKY3 (Figure S1) and WRKY4 proteins (Figure S2). The strong conservation of core residues around the WRKY domain region explains their evolutionary significance as the least disturbances might have occurred during their phylogenetic origin which revealed their crucial functional role.

### Functional sites identification

For the identification of functional sites found within the SlWRKY3 and SlWRKY4 sequences was analyzed using ExPASy-PROSITE (http://prosite.expasy.org/) to get the functional signatures sequences lying within the WRKY DNA binding domains. The PROSITE results revealed and confirmed the query protein sequences of both SlWRKY3 and SlWRKY4 belongs to the WRKY gene superfamily, and have retrieved the position of functional signature sequences from both N-terminal end (NTD) and C-terminal end (CTD) that constitutes the WRKY domain region occupying in the protein.

### Domain analysis

**Figure 3:**
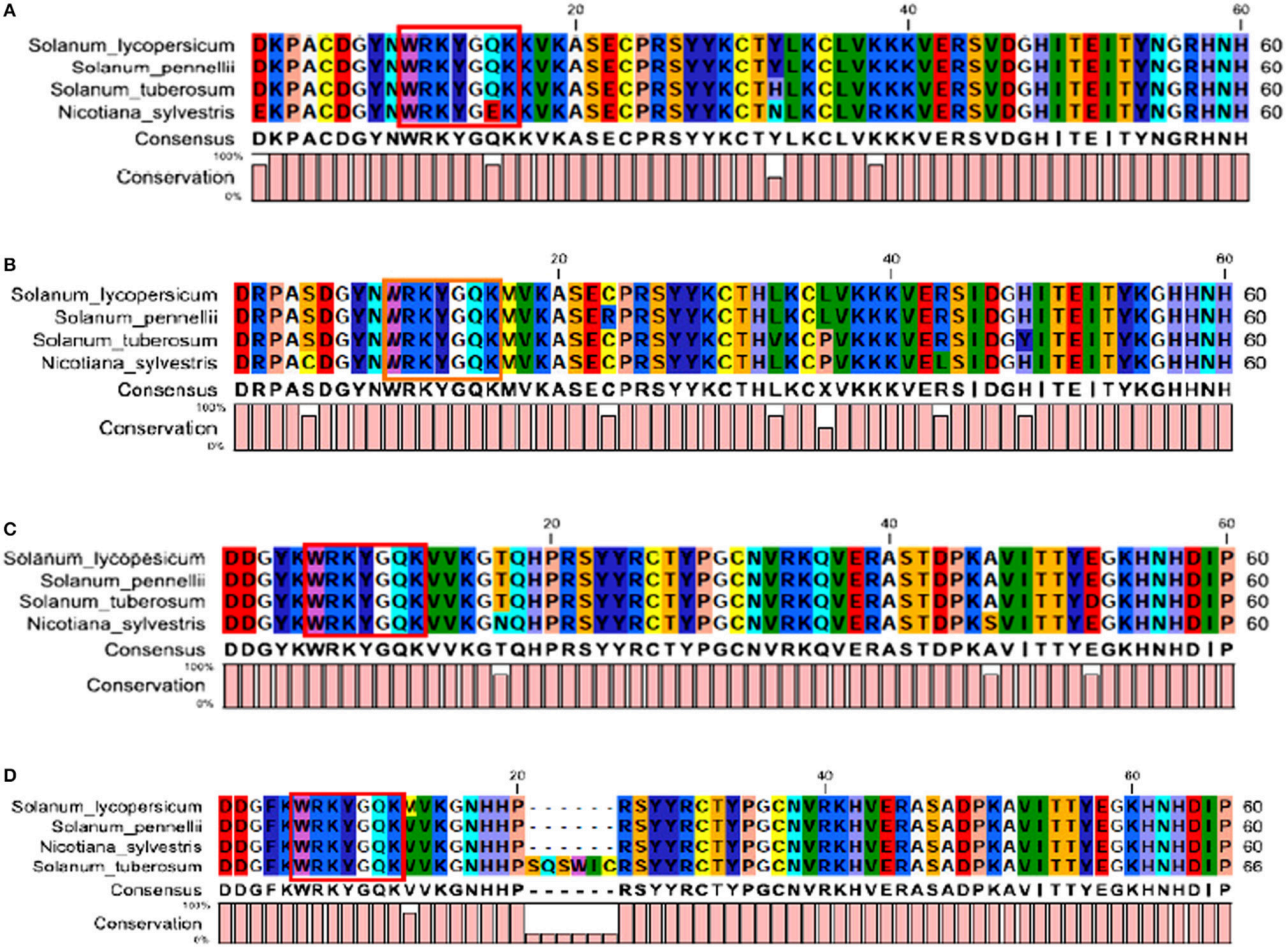
**(A)** The sequence alignment of the conserved functional WRKY domain region from N-terminal end in SlWRKY3, **(B)** N-terminal end of WRKY4, **(C)** C-terminal end of SlWRKY3, and **(D)** C-terminal end of SlWRKY4.

The InteProScan results revealed the presence of two WRKY DNA binding domains (at both N-terminal and C-terminal end) in each of SlWRKY3 and SlWRKY4. In SlWRKY3 the N-terminal domain lies (NTD) from 210 to 275 amino acid residues in which the core functional signature sequences were found to exist between Asp^218^ and Pro^275^ whereas the C-terminal domain (CTD) lies from 372 to 446 amino acid residues having functional signature sequences lying in between Ser^381^ to Pro^446^ (Figure S3). In SlWRKY4, also two domains were identified, with the N-terminal domain lying from Ser^208^ to Pro^272^ having functional sequences in the same region and the other C-terminal domain that lies from Glu^370^ to Pro^444^ containing the signature sequences lying in between Ser^379^ and Pro^444^ (Figure S4). The multiple sequence alignment done for the functional domain region (comprised of 60 amino acids) across all the members showed the strong conservation of residues for WRKY3 and WRKY4 both at the N-terminal (Figures 3A,B) and C-terminal end (Figures 3C,D). The two domains were found to be situated distantly and separated from each other by a long stretch of amino acids. The structure of zinc finger residues at both NTD and CTD were found to be different as in SlWRKY3 and SlWRKY4 the composition of zinc finger at the NTD were found to be C-X6-C-H27-H-X-H whereas, the CTD zinc finger structure in both SlWRKY3 and SlWRKY4 were found to have C-X4-C-X23-H-X-H type zinc finger structure.

### Motif analysis

**Figure 4:**
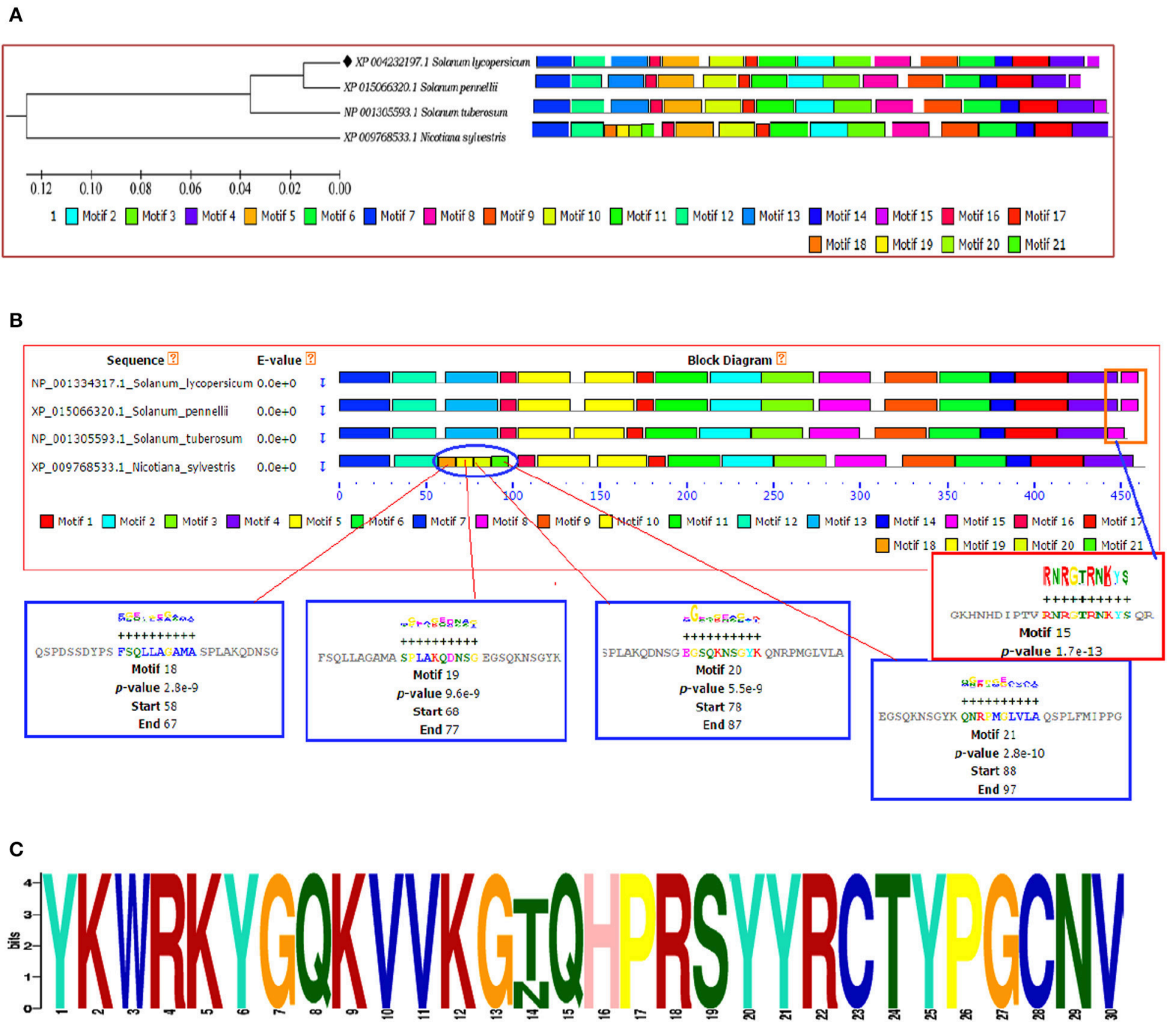
**(A)** The motif scan analysis represented along with phylogenetic tree showing the distribution and presence or absence of common and uncommon motifs found in *Solanum lycopersicum, Solanum pennellii, Solanum tuberosum*, and *Nicotiana sylvestris* discovered through MEME and MAST results. **(B)** The BLOCK diagram showing the sequence of the discovered motifs for SlWRKY3. The red arrows indicates the presence of uncommon motifs (motif 18, 19, 20, and 21) found in *Nicotiana sylvestris* and absent from other members, the red square indicate the presence of common motif (motif 15) that is absent from *N. sylvestris*. **(C)** The sequential logo of the motif 1 showing consensus WRKY sequences and present in all the representative members of tomato family.

**Figure 5:**
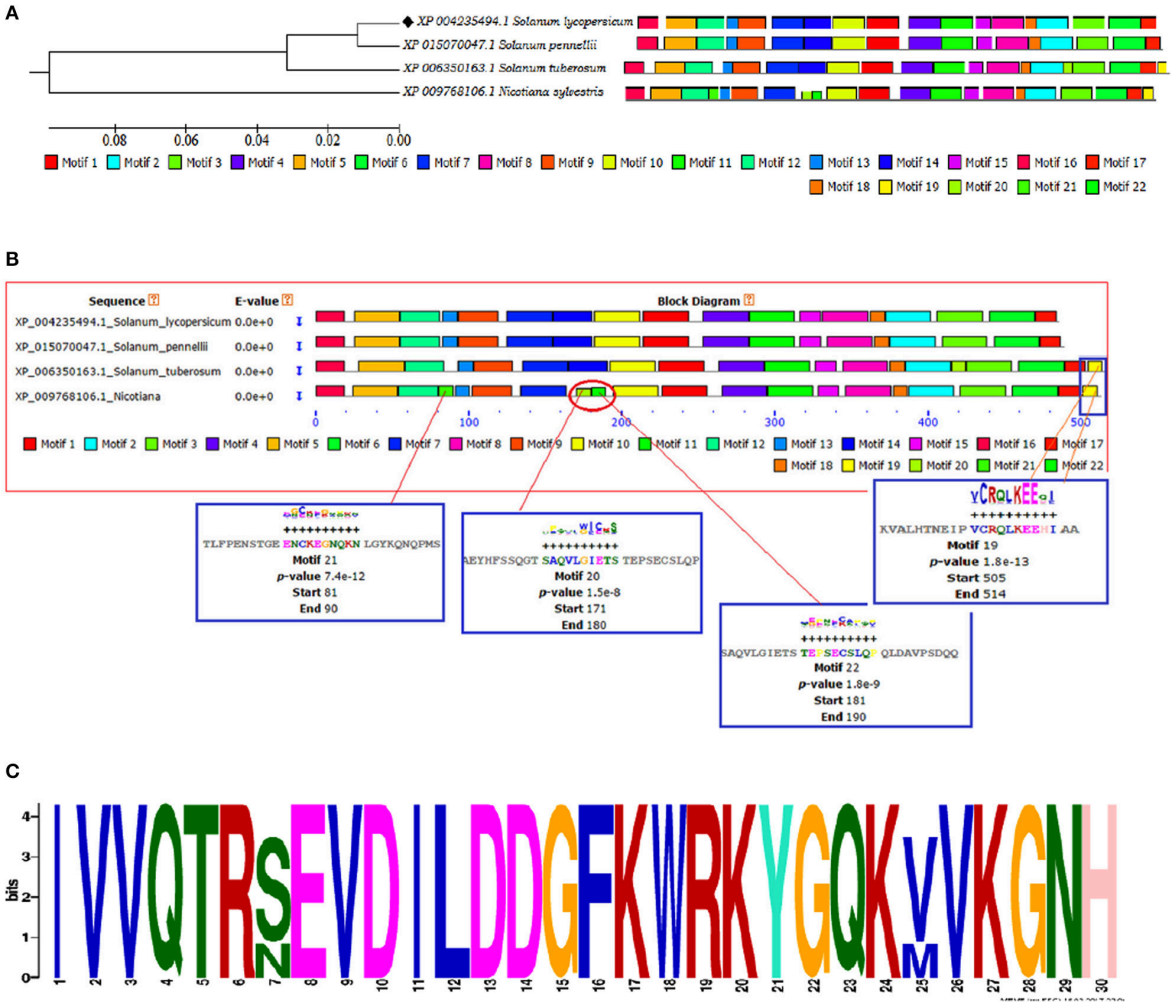
**(A)** The motif scan analysis represented along with phylogenetic tree showing the distribution and presence or absence of common and uncommon motifs found in *Solanum lycopersicum, Solanum pennellii, Solanum tuberosum*, and *Nicotiana sylvestris* discovered through MEME and MAST results. **(B)** The BLOCK diagram of the discovered motifs for WRKY4 protein in tomato. The red arrows indicates the uncommon motifs found exclusively in *Nicotiana sylvestris* and showing the distribution of motif 19 absent from *Solanum lycopersicum* and *Solanum penellii*. **(C)** The sequential logo of the motif 2 showing consensus WRKY sequences and present in all the representative members of tomato family.

The identification and characterization of transcriptional binding sites is central to annotating genomic regulatory regions. The occurrence of statistically significant transcription factor binding sites (motifs) in a DNA sequence may help in understanding the gene regulatory network (Zheng et al., 2017). Moreover, the occurrence of these functionally conserved network elements (motifs) may reflect their potential functions as some of them have been reported to act as nuclear localization signals assist in phosphorylation or other may provide calmodulin binding sites or protein dimerization initiators characteristic for leucine zippers (LZs; Grzechowiak, 2014). The MEME motif scan analysis revealed the commonalities in the distribution of motifs across all the members for both WRKY3 and WRKY4 proteins. The motif distribution analyzed in the form of phylogenetic tree for WRKY3 (Figure 4A) and WRKY4 (Figure 5A) cluster *S. lycopersicum* and *S. pennellii* into one group with *S. tuberosum* phylogenetically more closer to the above two rather than *N. sylvestris*. As the multiple sequence alignment done for the full length WRKY3 and WRKY4 protein sequences showed the conservation of maximum residues along the full length protein sequences, which also reflects from the distribution of motifs across all the members. However, the motif scan results predicted the importance of motifs that constitute the WRKY domain region. The statistical significance of motif finding can be evaluated in terms of their *p*- and *E*-value. The *E*-Value given in the BLOCK diagramme is conservative value and represents the statistical significance of each motifs and their likewise occurrence whereas the *p*-value represent an estimate of how well each occurrence matches the motif. In our results, the N-terminal WRKY domain (NTD) from SlWRKY3 was represented by the motif 2 (KPACDGYNWRKYGQKKVKASECPRSYYKCT; *p*-value 8.0e – 41) and the C-terminal Domain (CTD) was constituted by the motif 1 (YKWRKYGQKVVKGTQHPRSYYRCTYPGCNV; *p*-value 2.7e – 41) have least and significant *p*-values with more conserved CTD. In contrast, the WRKY domain region occupied in the NTD of SlWRKY4 was constituted by motif 1 (IVVQTRSEVDILDDGFKWRKYGQKMVKGNH; *p*-value 1.7e – 41) was found to be more conserved (least site *p*-value; Schmutz et al., 2010) rather than the CTD (DGYNWRKYGQKMVKASECPRSYYKCTHVKC; *p*-value 7.4e – 39). The propensity of finding a particular amino acid residue at a defined position in any motif could be revealed by the size of the alphabets that represent the amino acids. The first five motifs identified in WRKY3 and WRKY4 protein as discovered using MEME and MAST were shown in (Figures S5, S6). The motif scan analysis revealed the more commonalities as observed in *Solanum lycopersici, Solanum pennellii*, and *Solanum tuberosum* (motif 15: RNRGTRNKYS; *p*-value 1.7e – 13). Since, multiple sequence alignment for the full length WRKY3 and WRKY4 protein sequences revealed the conservation of residues which also reflects from our motif distribution results. The substitution of one or more amino acid residues with other residues resulted into the sequence divergence and thus leads into separate group or WRKY member. Furthermore, the presence of additional domains or uncommon motifs explains their divergence in the same group. However, the presence of these additional structural motifs is conserved among the different subsets of a particular WRKY family member as each motif is unique for a certain group. In our results, the *Nicotiana sylvestris* WRKY3 (NsWRKY3) showed the presence of four additional motifs including the motif 18 (FSQLLAGAMA; *p*-value 2.8e – 9), motif 19 (SPLAKQDNSG; *p*-value 9.6e - 9) along with motif 20 (EGSQKNSGYK; *p*-value 5.5e - 9) and motif 21 (QNRPMGLVLA; *p*-value 2.8e – 10). The presence of additional motifs and loss of common motif 15 (RNRGTRNKYS; *p*-value (1.7e – 13) in *N. sylvestris* (Figure 4B), that leads into sequence divergence and formed a separate cluster in the phylogenetic tree. Similarly, in NsWRKY4 the additional uncommon motifs found were the motif 20 (SAQVLGIETS; *p*-value 7.9e – 12), motif 21 (ENCKEGNQKN; *p*-value 7.4e – 12 and motif 22 (TEPSECSLQP; *p*-value 1.8e – 9). The motifs 19 and 20 were found to be present in both *N. sylvestris* and *S. tuberosum* but absent from *S. lycopersicum* and *S. penellii* which indicates the common evolutionary origin of *S. lycopersicum* from its wild homolog (*S. pennellii)* and the sequence divergence from other two members (potato and tobacco) from the same family (Figure 5B). However, the formation of additional motifs (motif 21 and motif 22) were solely found in *N. sylvestris* and therefore, grouped in separate cluster. The sequential logo digramme for the motif 1 having sequences lying in the WRKY3 DNA binding domain have been represented (Figure 4C) and for motif 2 having sequences lying in the WRKY4 DNA binding domain have been represented (Figure 5C). These sequences showed high conservation value among all the four members as indicated by the height of the alphabetical letters.

### Protein-protein interaction network

**Figure 6:**
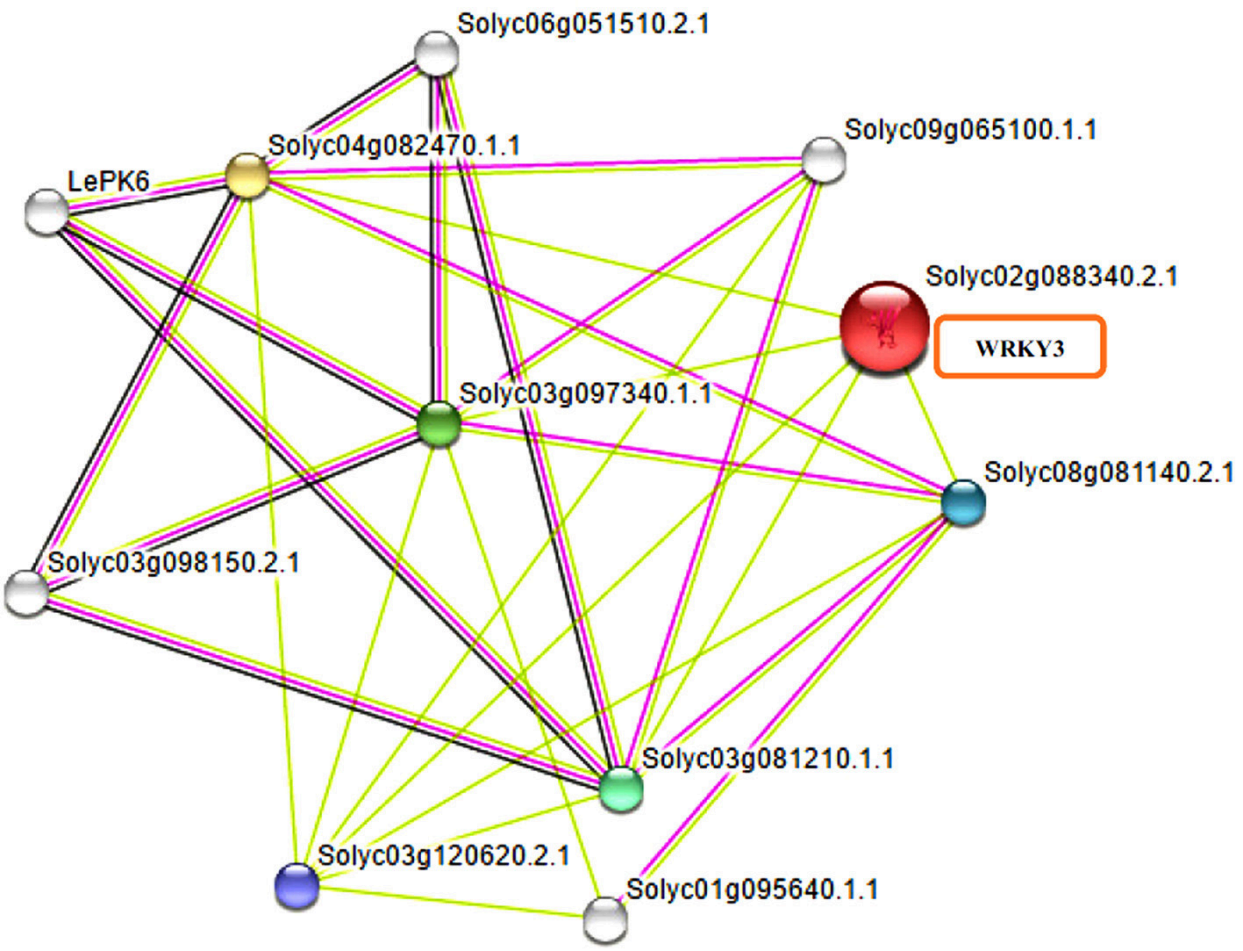
**Functional interactive associative network of tomato WRKY3 with other protein family members as found on STRING server datasets at medium confidence leveland represented in multifaceted way where the color nodes describe query proteins and first shell of interactors whereas white nodes are second shell of interactors**. The large node size represent characterized proteins and smaller nodes for uncharacterized proteins.

WRKY transcription factors have been reported to interact with many other proteins playing significant role in signaling, transcription and chromatin remodeling (Chi et al., 2013). It is now well-reported that WRKY transcription factors form both homo and heterocomplexes and this could be achieved through interaction of WRKY proteins from group IIa with each other through leucine zipper motifs (Cormack et al., 2002; Xu et al., 2006) interaction of group III WRKY TFs (Besseau et al., 2012) and heterodimerization of members of group IIb. The homo and hetero dimers formed by *Tamarix hispida* WRKY4 (ThWRKY4) with ThWRKY2 and ThWRKY3 are involved in mediating various abiotic stress responses (Wang L. et al., 2015). These protein-protein interactions observed between WRKY members and WRKYs with other protein partners of different families have elucidated the pathways involved in complex web signaling. Therefore, revealed the important informations regarding the mode of function and regulation of different WRKY members. Furthermore, the slight variations found in WRKY DNA binding domain and the presence of other conserved motifs found in different WRKY subfamilies participate in protein-protein interactions and mediate complex functional interactions. The STRING database provides critical assessment and integration of protein-protein interactions, including direct (physical) as well as indirect (functional) associations. The results obtained through STRING server have shown the different interacting partners for tomato WRKY3 (Figure 6) at medium confidence level by selecting the default custom values of 10 interactors from first and at least five protein interactors from second shell of interactions. The tomato WRKY3 (Solyc02g088340.2.1) was found to have in interaction with several proteins with maximum interaction score values (0.699), with TRANSPARENT TESTA GLABRA1-like (Solyc03g081210.1.1; NCBI Protein ID XP_004235332). Others include homeobox leucine zipper protein GLABRA2 like (Solyc03g120620.2.1; NCBI protein ID XP_004235676), that was reported to be strongly expressed in the trichomes throughout their development, in the endothelium of developing seeds, other layers of the seed coat, and also in the atrichoblasts of developing roots (Johnson et al., 2002), GLABRA3 like transcription factor (Solyc08g081140.2.1; NCBI protein ID NP_001333930; a basic helix loop type) and WD repeat-containing protein LWD1-like (Solyc04g082470.1.1; NCBI Protein ID XP_004238433) that function as multi subunit ring-finger type E3 Ubiquitin ligases (E3), playing an important role in plant defense response.

**Figure 7:**
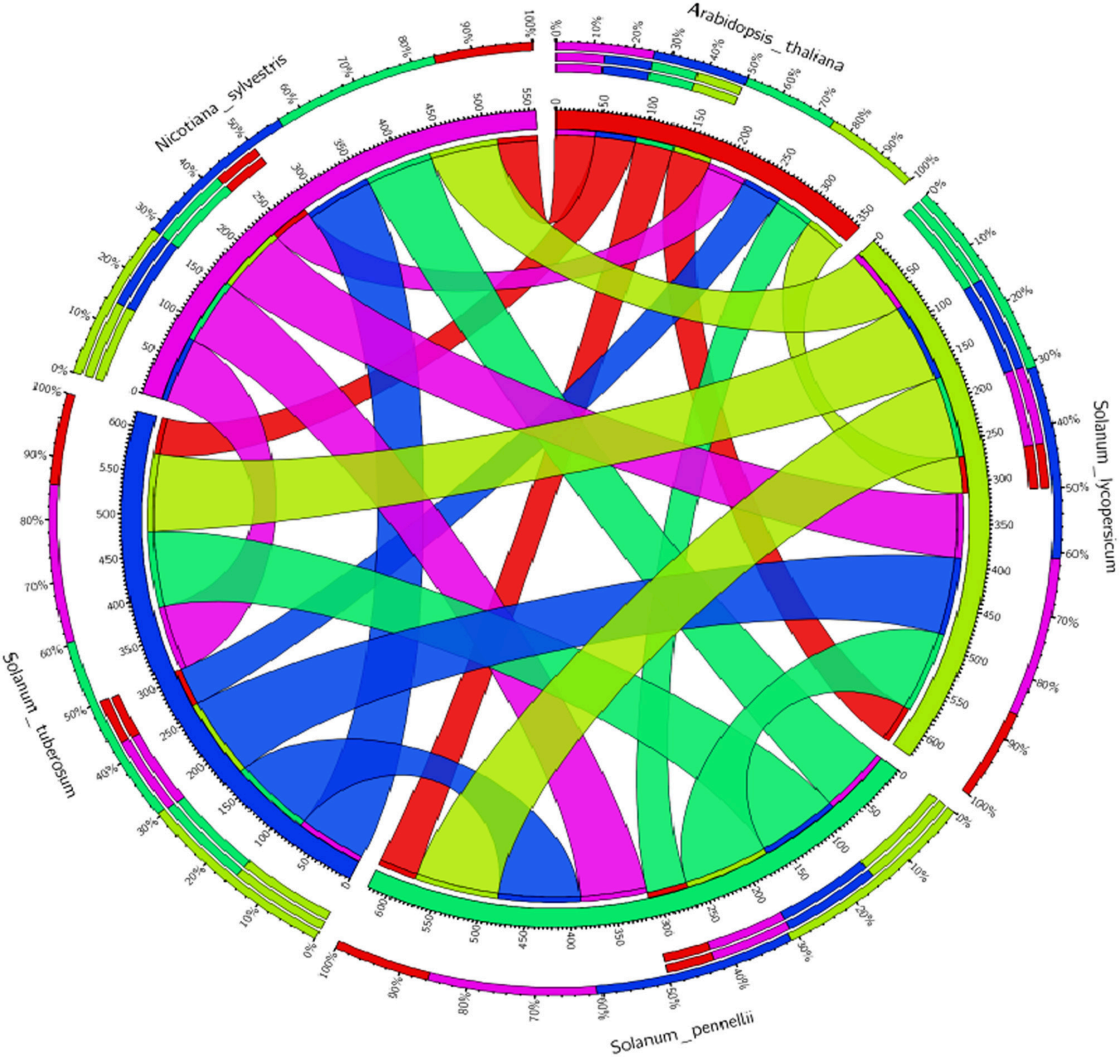
**Comparative analysis of the tomato WRKY genes (SlWRKY3 and SlWRKY4) with the others members of family Solanaceae along with model *Arabidopsis thaliana* to investigate the similarities and differences**. The circular map generated based on percentage similarity matrices (obtained through phylogenetic clustering using ClustalW) and have been visualized through Circos software.

In contrast, we do not find any interacting partners for tomato WRKY4 at high confidence level based on the current available datasets at STRING server. However, the tomato homologs of *Arabidopsis* showed SUMO1 interacting with WRKY4 at high confidence level. The interacting score values obtained for SlWRKY3 with different proteins have been listed in (Table S1) and those for SlWRKY4 (Table S2). For further validation of our results, we have used the Predicted Tomato Interactome Resources (PITR) and analyzed all the possible interactive partners for tomato WRKY3 and WRKY4 (Figure S7) using *Arabidopsis thaliana* protein interactome database (AtPID) since, *Arabidopsis* shares the highest evolutionary conservation with tomatoes (Yue et al., 2016). It has been suggested that the functional conservation itself could be employed for interactome analysis as the conserved proteins are likely to have same functions. Therefore, the functional knowledge and interaction network can likely be transferred to different species having orthologous relationship with previous partners (Sun and Kim, 2011).

### Structural modeling of WRKY domain

**Figure 8:**
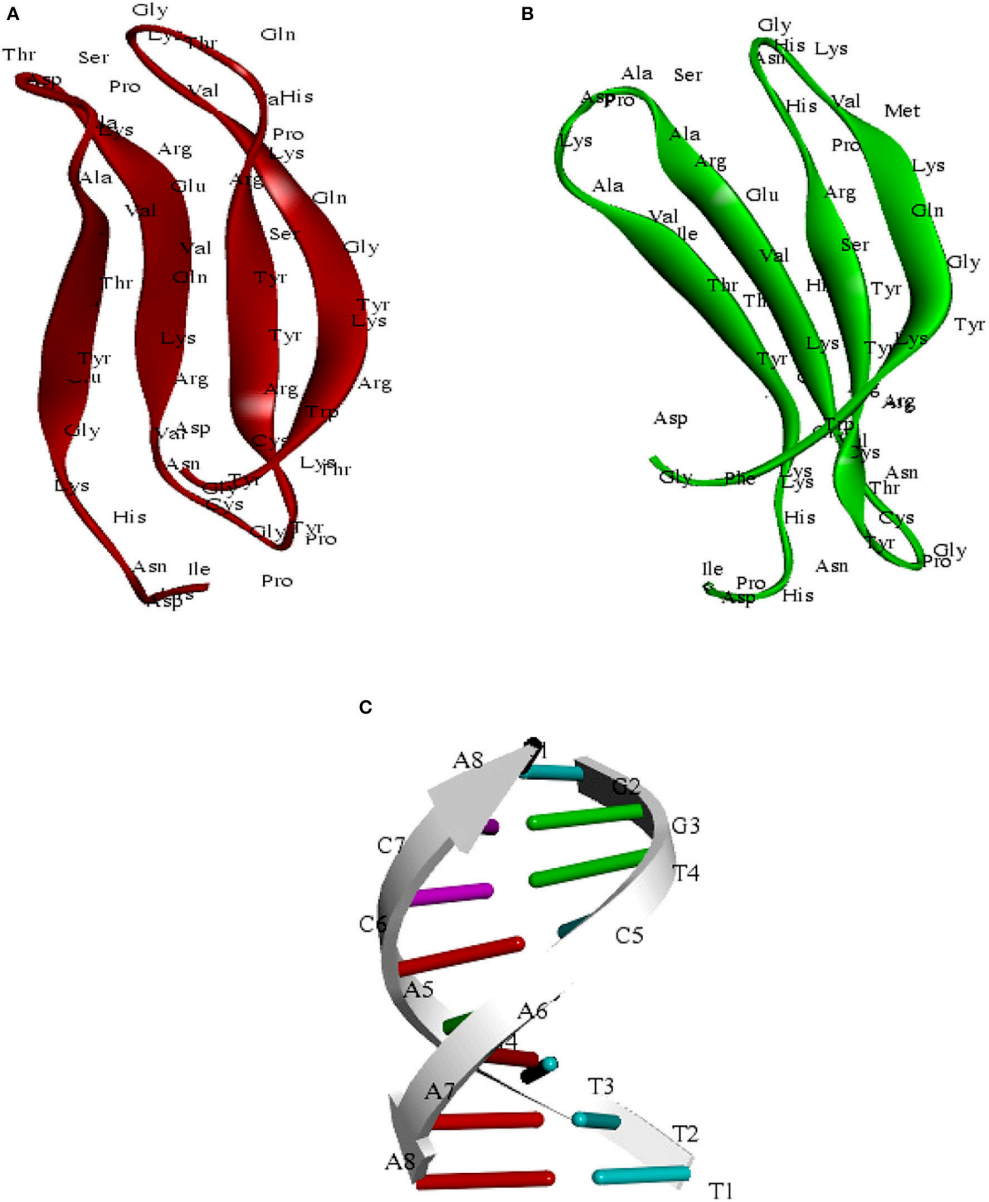
**(A)** Predicted structure of the C-terminal WRKY3 domain visualized through the Discovery Studio 3.0. **(B)** Structure of the C-terminal WRKY4 domain **(C)** structure of the W-Box (TTTGACCA)DNA sequence.

We have modeled the structure of C-terminal domain (CTD) for both SlWRKY3 and SlWRKY4 The appropriate template was chosen based on sequence similarity, residue completeness, and crystal resolution. The total five models were generated by DS Modeller [Each for SlWRKY3 (Table 1) and SlWRKY4 (Table 2)] and the model having the least RMSD with respect to trace (Cα atoms) of the crystal structure of the template was selected for further interactions. The minimum electrostatic energy is the most important parameter that predicts the protein structural stability and model reliability (Pokala and Handel, 2005). In our results, the modeled C-terminal domain for SlWRKY3 (Figure 8A) and SlWRKY4 (Figure 8B) protein stability was good enough based on the total calculated electrostatic energy (minimum). The specifically recognized W-box sequence (TTTGACCA) was used for DNA modeling using DNA sequence to structure tool http://www.scfbio-iitd.res.in/software/drugdesign/bdna.jsp (Arnott et al., 1976; Figure 8C). We have submitted our predicted models at the PMDB. The PMDB assigned the PMDB IDs for our submitted PDB structures of SlWRKY3 WRKY DNA binding domain (PM0080567) and SlWRKY4 (PM0080566) have been visualized in (Figure S8).

**Table 1:** **Different energy parameters for the modeled SlWRKY3 protein measuring their structural stability on the basis of Discrete optimized protein energy (DOPE Score), PDF physical energy, and PDF total energy**.

**Model name**	**PDF total energy**	**PDF physical energy**	**DOPE score**
**PREDICTED MODEL SCORES SlWRKY3**			
MODEL1(M0001)	267.4683	159.07849	–4106.107422
MODEL3(M0003)	274.2886	161.134994	–4093.040039
MODEL4(M0004)	284.0109	164.137915	–4076.419189
MODEL5(M0005)	310.2852	165.89298	–4058.052002
MODEL2(M0002)	329.7176	171.88393	–4100.641602

*The DOPE model score is designed for selecting the best structure from a collection of models built by MODELLER and the best model can be judged by either low dope score or low mol PDF*.

**Table 2:** **PDF total energy, PDF Physical energy and DOPE score for modeled SlWRKY4**.

**Model Name**	**PDF Total Energy**	**PDF Physical Energy**	**DOPE Score**
**PREDICTED MODEL SCORES WRKY4**			
MODEL2(M0002)	264.1011	161.4889192	−4059.075928
MODEL5(M0005)	278.8530	159.67754	−4069.148193
MODEL3(M0003)	291.3958	164.255614	−4109.856934
MODEL4(M0004)	302.1431	159.61964	−4057.975342
MODEL1(M0001)	318.5243	200.18065	−4150.335938

*Models are arranged on the basis of their structural stability and satisfying the energy parameters and obtained after loop refinement*.

### Superimposition results

**Figure 9:**
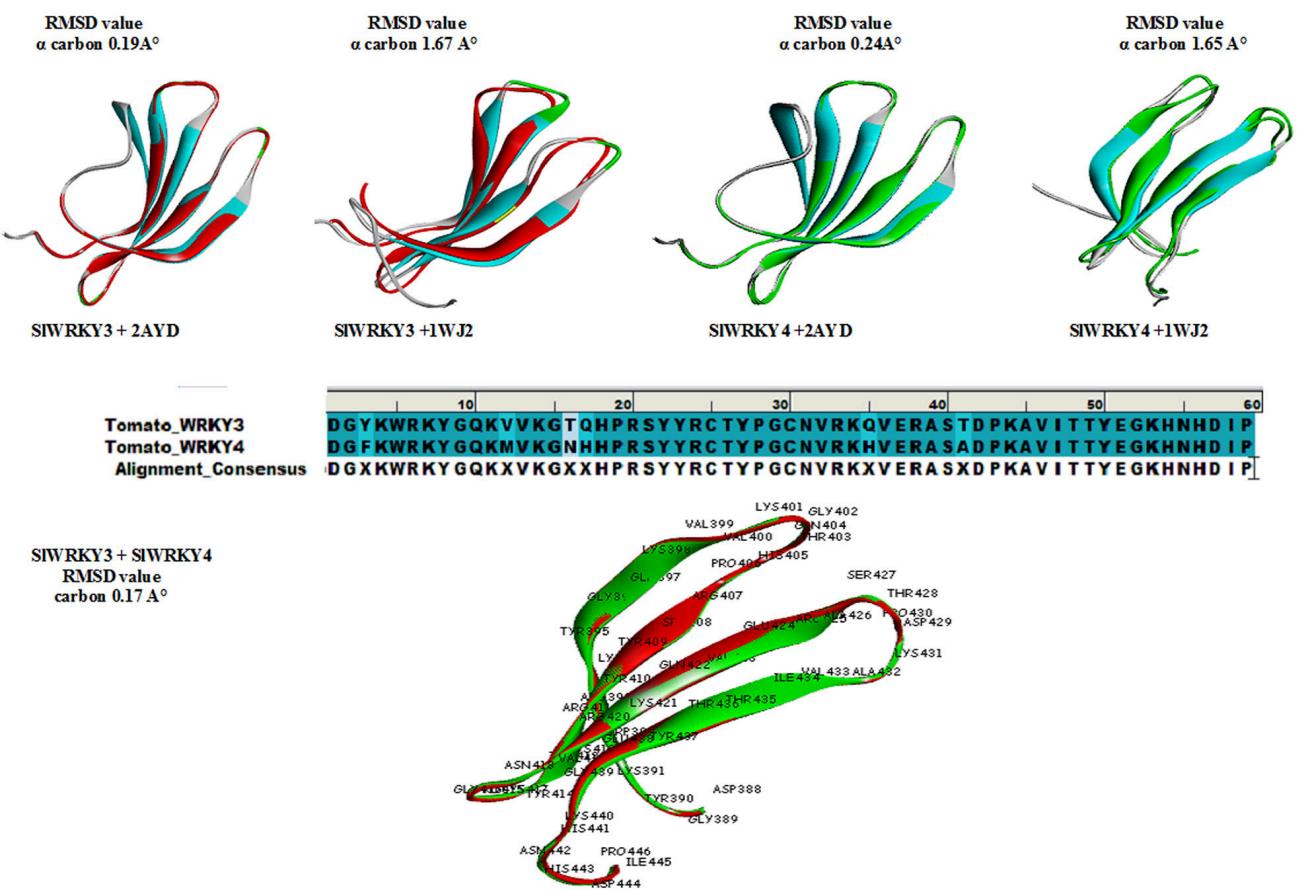
**Superimposition results represented with their respective global and local RMSD-values showing the structural conservation of WRKY domain**. Superimposition revealed that both SlWRKY3 and SlWRKY4 were found to be structurally more closer to their template 2AYD rather than other template 1WJ2 as evident from fluctuations in their RMSD values. Sequence alignment between SlWRKY3 and SlWRKY4 predict the synonymous (deep blue) and non-synonymous substitutions (light blue) color. We have labeled the residues only from tomato WRKY3 to show different residues with respect to their topology and their sitewise probable occurrence in protein secondary structure.

The protein structure is 3–10 times more conserved than sequences (Illergård et al., 2009). It has been well-suggested that the evolution of proteins took under strong structural constraints that results into the fact that proteins drifted apart overtime due to divergent evolution may still exhibit structural resemblance despite of having no sequence similarity (Panchenko and Madej, 2005). The predicted domain structures SlWRKY3 and SlWRKY4 were superimposed over each other for comparing their topological (structural) details (Figure 9). The sequences were aligned using BLOSUM 62 matrix having gap penality 10.0 with extend penalty 0.5. The superimposition results shows six amino acid residues were found to be substituted in between SlWRKY3 and SlWRKY4 with covering the overall sequence similarity of 89.8% (53/59) and 93.2% (55/59) positives. The differences between RMSD-value for the predicted WRKY3 and WRKY4 models were found to be 0.17 A° alpha carbon atom and 0.19 A° for the back bone atoms. The structural alignment from SALIGN web server predicted the maximum similarity score of the target protein with its template structure. The structural alignment for predicted model and template selected showed the maximum similarity score of SlWRKY3 and SlWRKY4 with 2AYD than 1WJ2. This was also confirmed from RMSD-values as when the predicted modeled SlWRKY3 was aligned with template 2AYD the global and local RMSD-values were 0.19 A° alpha carbon and 0.27 A° around backbone atoms whereas when the same template was aligned with SlWRKY4 the RMSD-value for alpha carbon atom was 0.24 A° and 0.28 A° around backbone atoms. In contrast, when the SlWRKY3 was superimposed over the template 1WJ2 the calculated RMSD-values were found to be 1.67 A° alpha carbon and 1.63 A° around the backbone atoms. Similarly, the superimposition of SlWRKY4 over 1WJ2 leads into the global and local RMSD-values 1.65 A° alpha carbon and 1.62 A° for the backbone atoms. These results concluded the conservation of WRKY domain structues along with sequence similarity across the divergent WRKY members. However, the sequence alignment of the functional CTD of SlWRKY3 and SlWRKY4 revealed some synonymous or non-synonymous substitutions (light blue) which distinguished the SlWRKY3 domain from SlWRKY4. Furthermore, the strong sequential homology was observed around the consensus motif KWRKYGQK in both SlWRKY3 and SlWRKY4 underlying in the WRKY domain. We have found amino acid substitutions at some positions such as Phe^388^, Met^397^, Asn^401^, His^402^, His^420^, and Ala^426^ in SlWRKY4, when compared to SlWRKY3, where Tyr^388^, Val^397^, Thr^401^, Asn^402^, Asn^420^, and Thr^420^ were present.

### Model evaluation

Five sets of models each for SlWRKY3 and SlWRKY4 were generated employing the satisfaction of spatial restraint using Modeller Discovery Studio Client 3.0. The three dimensional (3D) model is obtained by optimally satisfying all the spatial restraints derived from the alignment and expressed as probability density functions (PDFs) for the features restrained. Discrete optimized protein energy (DOPE) is a statistical potential used to assess the homology model in protein structure prediction (Eramian et al., 2006), and is based on an improved reference state that corresponds to non-interacting atoms in a homogeneous sphere with the radius dependent on a sample native structure thus it accounts for the finite and spherical shape of the native structures (Shen and Sali, 2006). DOPE is implemented in python and is run within the MODELLER environment (John and Sali, 2003). The values of DOPE score for five models generated models were reported in arranged on the basis of their stability satisfying all the essential energy parameters. Out of these five models the model having lowest DOPE score value was selected as final model.

### Model validation

**Figure 10:**
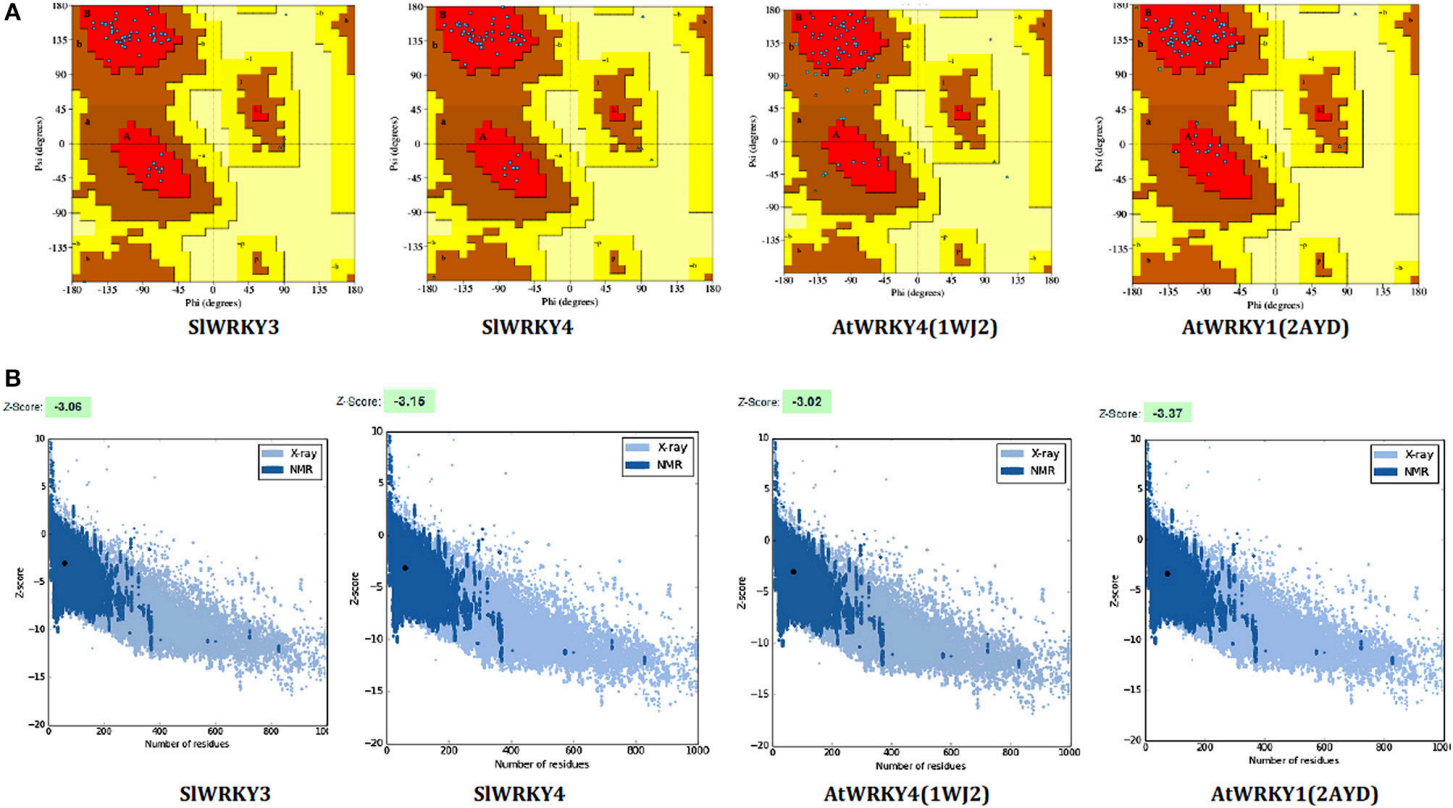
**Qualitative evaluation of the predicted model using PROCHECK and ProSA analysis. (A)** The stereo chemical spatial arrangement of amino acid residues in the predicted models (SlWRKY3 and SlWRKY4) as computed with the PROCHECK server and were compared with experimentally resolved protein structures (1WJ2 and2YD). Most favored regions are colored red, additional allowed, generously allowed, and disallowed regions are indicated as yellow, light yellow, and white fields, respectively. **(B)** Qualitative evaluation using ProSA webserver, which generates a plot measuring the structural error at each residues in the protein and calculate the overall score for quality measurement. The ProSA score for SlWRKY3 and SlWRKY4 were found closer to the native structures.

Ramachandran plot analysis was done using RAMPAGE and PDBSum servers and based on RAMPAGE statistics it was found that 100% of the amino acid residues were observed in the most favored regions against the ~98.0% expected, and 0.0% residues were found in the allowed region against the ~2.0% expected values for both SlWRKY3 and SlWRKY4 (Figure S9). We have compared our predicted models with the template proteins (NMR derived solution structure of C-terminal domain of AtWRKY4 PDB ID:1WJ2) and the X-ray determined crystal structure of C-terminal domain of AtWRKY1 (PDB ID: 2AYD). The CATH server classified the protein SlWRKY3 and SlWRKY4 to exist as beta sheet type secondary structure, which is further confirmed by results of the Volume, Area, Dihedral Angle Reporter (VADAR) which evaluated the predicted model based on quantitative parameter. The VADAR results predicted the topological conformation that existed for SlWRKY3 and found to have observed values of 0 (0%) helix, 33 (55%) beta, coil 26 (44%), and turns 12 (20%) with the observed mean H bond energy (−2.2 *SD* = 0.7) against the expected values of −2.0 (*SD* = 0.8). In contrast, for SlWRKY4 we got minimum deviation with 0 (0%) helix, 32 (54%) beta, coil 27 (45%), and turns 12 (20%) with the observed mean H bond energy (−2.2 *SD* = 0.7) against the expected values of −2.0 (*SD* = 0.8). The qualitative evaluations of the modeled proteins both SlWRKY3and SlWRKY4 were further validated and verified using various single model methods including PROCHECK (Laskowski et al., 1993), Qualitative Model Energy Analysis (QMEAN; Benkert et al., 2009), Protein Structural Analysis (ProSA; Wiederstein and Sippl, 2007), Resolution by Proxy (ResProx; Berjanskii et al., 2012). Based on PROCHECK analysis of PDBSum results it was found that total 100.0% of the residues occurred in the most favored regions (A, B, L), 0.0% residues were found in additional allowed regions (a, b, l, p) with 0.0% residues were in generously allowed regions (~a, ~b, ~l, ~p) and no residue was located in the disallowed regions (XX) (Table 3). The PROCHECK results for SlWRKY3 and SlWRKY4 were shown (Figure 10A). The qualitative assessment of the predicted model proteins were also compared based on PROCHECK results with template proteins 1WJ2 and 2AYD. The PROCHECK based assessment determines the stereochemical quality of the modeled protein, such as main-chain bond lengths and bond angles. A good quality model is expected to have over 90% residues in the most favored regions [A, B, L]. Further, ProSA server was used for the recognition of errors in experimental and theoretical models. The *z*-score evaluates the overall model quality and measures the deviation of the total energy of the structure with respect to an energy distribution derived from all the random conformations. ProSA evaluates the model packing by estimating the probability for finding residues at specific distance and also evaluates the extent of interactions existed between the model and the solvent i.e., solvation. The sum of all these probabilities overall determine and evaluate the reliability and quality of the generated model. In case of close template, C-terminal domain of AtWRKY4, the evaluated *Z*-score was −3.02 (1WJ2) and −3.37 for C-terminal WRKY domain of AtWRKY1 (template 2AYD) while in case of our computational generated/predicted model the *Z*-score was –3.02 (SlWRKY3) and −3.15 (SlWRKY4; Figure 10B). ProSA score revealed that template and target scores were very close to each other with minimum structural error difference. The above results indicate that the stereo chemical qualities of the protein structure coordinates are reliable. The predicted protein models were found to be good enough based on both qualitative and quantitative parameters. QMEAN (Qualitative Model Energy Analysis) evaluates the model quality based on the major geometrical aspects relevant to the protein structures. These geometrical aspects are molecular descriptors of local geometry, solvation potential, and secondary structure specific distance-dependent pairwise residue-level potential, for assessment of long-range interactions (Benkert et al., 2009). QMEAN generates a composite score values based on these descriptors for overall qualitative evaluation of the predicted models with theoretical available models (Figure S10 and Table S3).

**Table 3:** **Comparative score for the assessment of stereochemical quality (qualitative evaluation) for the modeled WRKY proteins (SlWRKY3 and SlWRKY4) and AtWRKY4 (template1) and AtWRKY1 (template2) and complex of AtWRKY4 with W-box DNA (template3)**.

**S.N**	**Protein Name**	**Qmean score**	***Z*-score**	**RESPROX**	**Most favored (%)**	**Additionally allowed (%)**	**Outlier residues (%)**
1.	SlWRKY 3 Domain (predicted model 1)	0.696	−3.06	1.03	100	0.0	0.0
2.	SlWRKY 4 Domain (predicted model 2)	0.706	−3.15	1.111	100	0.0	0.0
3.	C-terminal Domain of AtWRKY4 (PDB:1WJ2) (template1)	0.557	−3.02	2.274	89.9	5.8	4.3
4.	C-terminal Domain of AtWRKY1 (PDB: 2AYD) (template2)	0.811	−3.37	1.204	100	0.0	0.0
5.	Complex of C-terminal domain of AtWRKY4 and W- box DNA (PDB: 2LEX) (template3)	0.470	–2.94	2.793	77.0	14.8	8.2

*For protein to be of good quality this values ranges between 0 and 1.5 (ReSProx). In the modeled proteins most favored region occupies 100% and number of residues lies in outlier region and disallowed region covers 0.0% (PROCHECK) compared to template where number of residues in outlier and disallowed region covered significant residues*.

### DNA-protein interaction

**Figure 11:**
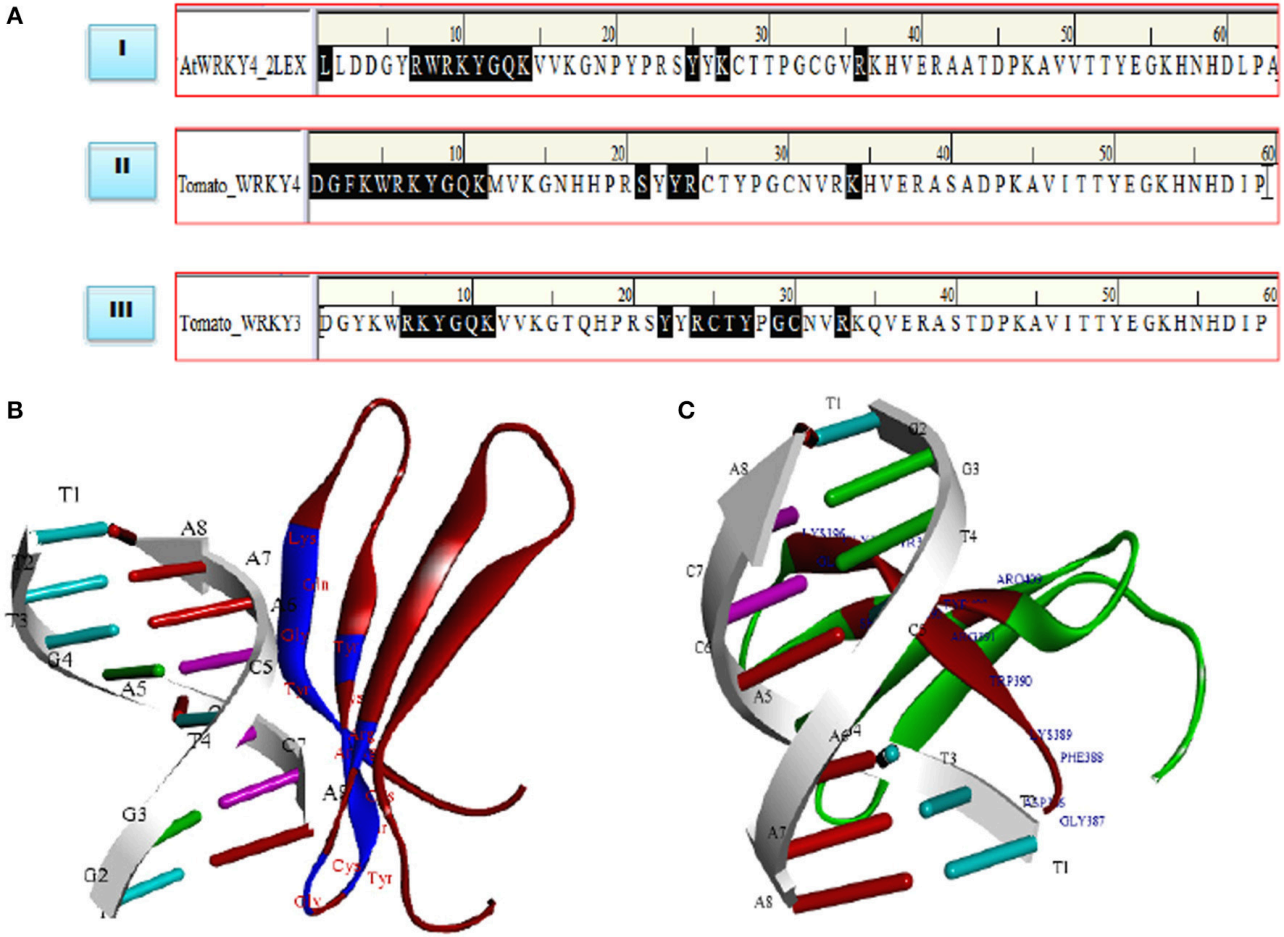
**(A)** Comparative evaluation of the docked complexes with experimentally solved structures. The NMR resolved solution structure of complex of the C-terminal WRKY domain of AtWRKY4 with W-box DNA. The residues LRWRKYGQK made interaction with W-box DNA and compared with our predicted complexes where (RKYGQK) along with residues forming zinc finger got involved in interaction (SlWRKY3) and similar residues KWRKYGQK with initial flanking sequences (SlWRKY4) participated in interaction with W-box. **(B)** Structure of the docked complex (SlWRKY3 with DNA) as visualized by Discovery Studio 3.0 **(C)** Structure of the docked complex (SlWRKY4 with DNA).

Molecular docking studies represent computational approaches toward exploration of possible binding mode of a ligand (SlWRKY3 and SlWRKY4) to a given receptor (DNA). Further, the 3D structure of specifically recognized W-box sequence was used for interaction with SlWRKY3 (Figure 11B) and WRKY4 domains (Figure 11C). For the experimental verification and validation of our DNA-protein interaction results obtained through computational approaches, we have analyzed the residues involved in DNA protein interaction from NMR determined solution structure of DNA-protein complex of C-terminal domain of AtWRKY4 in *A. thaliana* (2LEX; Figure 10A). The NMR-derived solution structure revealed that in AtWRKY4 the interacting residues that got involved in interaction with W-box DNA element were conserved WRKYGQK with the key residues including Leu^407^, Arg^413^, Trp^414^, Arg^415^, Lys^416^, Tyr^417^, Gly^418^, Glu^419^, Lys^420^, Tyr^431^, Lys^433^, and Arg^442^. We have docked the most reliable model (based on qualitative and quantitative energy parameters) both from SlWRKY3 and SlWRKY4 with the modeled W-box and the binding energy for the most stable complex (least energy values) was calculated. In case of SlWRKY3 the most stable complex docked have binding energy (*E*_*total*_ = −1297.56 Kcal/mol), whereas the SlWRKY4 the most stable complex was docked with binding energy (*E*_*total*_ = −1511.58 Kcal/mol; Figure S11). The DNA-protein interaction studies revealed the optimized conformation and the most possible relative orientation observed between DNA and protein so that the free energy of the overall system is minimized. The scoring functions are physics-based molecular mechanics force fields that estimate the energy of the pose. The low energy (most negative) predicts the stable system and therefore, the most possible binding interaction and stability of the docked complexes (Ritchie, 2003; Maria Antony Dhivyan and Anoop, 2012). The interacting amino acid residues from the AtWRKY4 (2LEX), SlWRKY3, SlWRKY4 involved in interaction with W-box DNA element have been shown (Figure 11A). The molecular docking studies revealed that SlWRKY3 binds with *cis*-DNA sequence through conserved RKYGQK and zinc finger motifs with the residues from zinc finger also participated in this interaction. The key residues that were involved were Arg^393^, Lys ^394^, Tyr^395^, Gly^396^, Glu^397^, Lys^398^, Tyr^409^, Arg^411^, Cys^412^, Thr^413^, Tyr^414^, Gly^416^, Cyst^417^, and Arg^420^. In contrast, SlWRKY4 binds through WRKYGQK with the help of initial flanking sequences. The residues involved in this interaction were Asp^386^, Gly^387^, Phe^388^, Lys^389^, Trp^390^, Arg^391^, Lys^392^, Tyr^393^, Gly^394^, Glu^395^, Lys^396^, Ser^406^, Tyr^408^, Arg^409^, and Lys^419^. In our results, the SlWRKY4 showed the similar residues of the WRKYGQK motifs involved in binding as those found in AtWRKY4. It is assumed that WRKY family members specifically bind to varying DNA motifs but disclose a common binding consensus core (Rushton et al., 1996; Eulgem et al., 2000; Ciolkowski et al., 2008; Yamasaki et al., 2012; Brand et al., 2013). The flanking sequences involved in DNA binding demonstrate the functional redundancy observed between different members and therefore, determine the specific regulation achieved by the WRKY members. The different genes in the genome of *Arabidopsis* have highly divergent structures but show strong conservation of WRKY domain (WRKYGQK.HXH.) (Eulgem et al., 2000). Furthermore, when a comparison of sequences designating only WRKY domain was made between *Arabidopsis*, tomato and *Capsella*, it was reported that the C-terminal domain (CTD) of tomato gene is more similar to the domains in *Arabidopsis* and *Capsella* than is the N-terminal domain (Rossberg et al., 2001) which also supports our results of DNA- protein interaction studies.

### Gene ontology enrichment analysis

**Figure 12:**
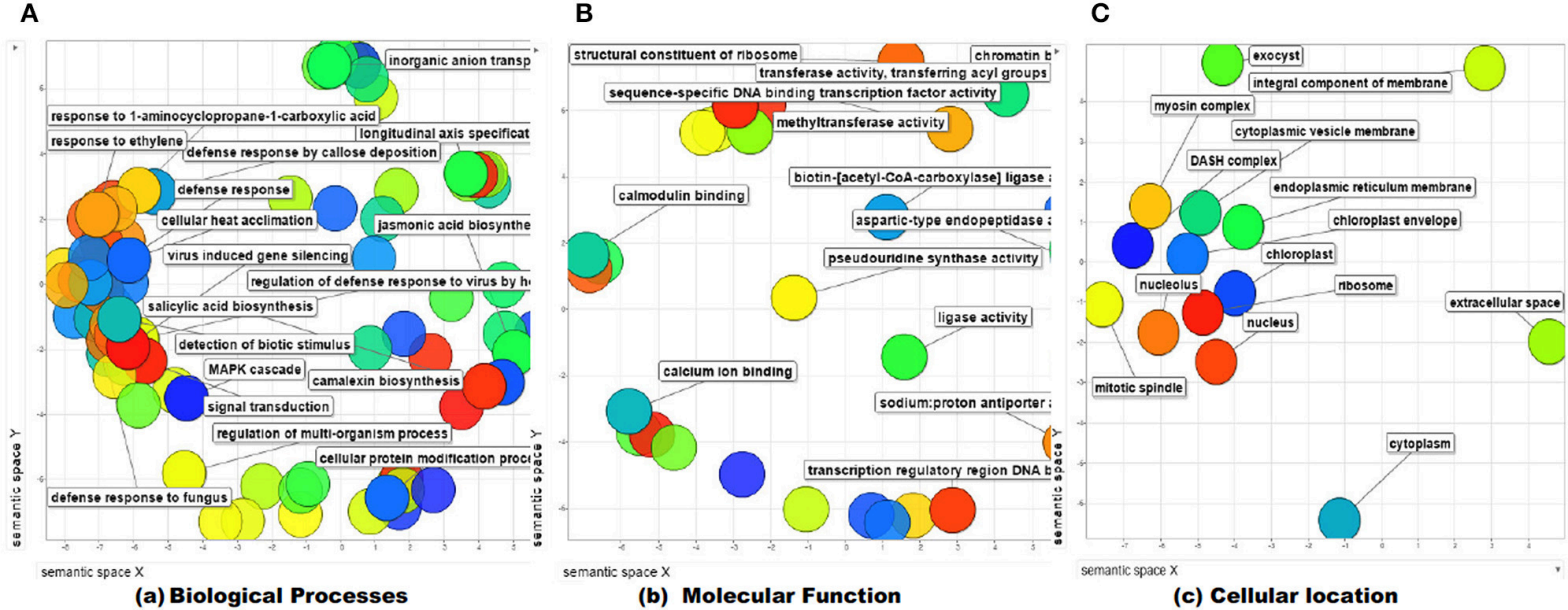
**Gene ontology enrichment analysis using ReviGO web server**. The functional and significant GO terms were shown on scattered plot digramme using hypergeometric test distribution in terms of their controlled functional vocabularies (**A**, biological process; **B**, molecular function; and **C**, cellular processes involved).

**Figure 13:**
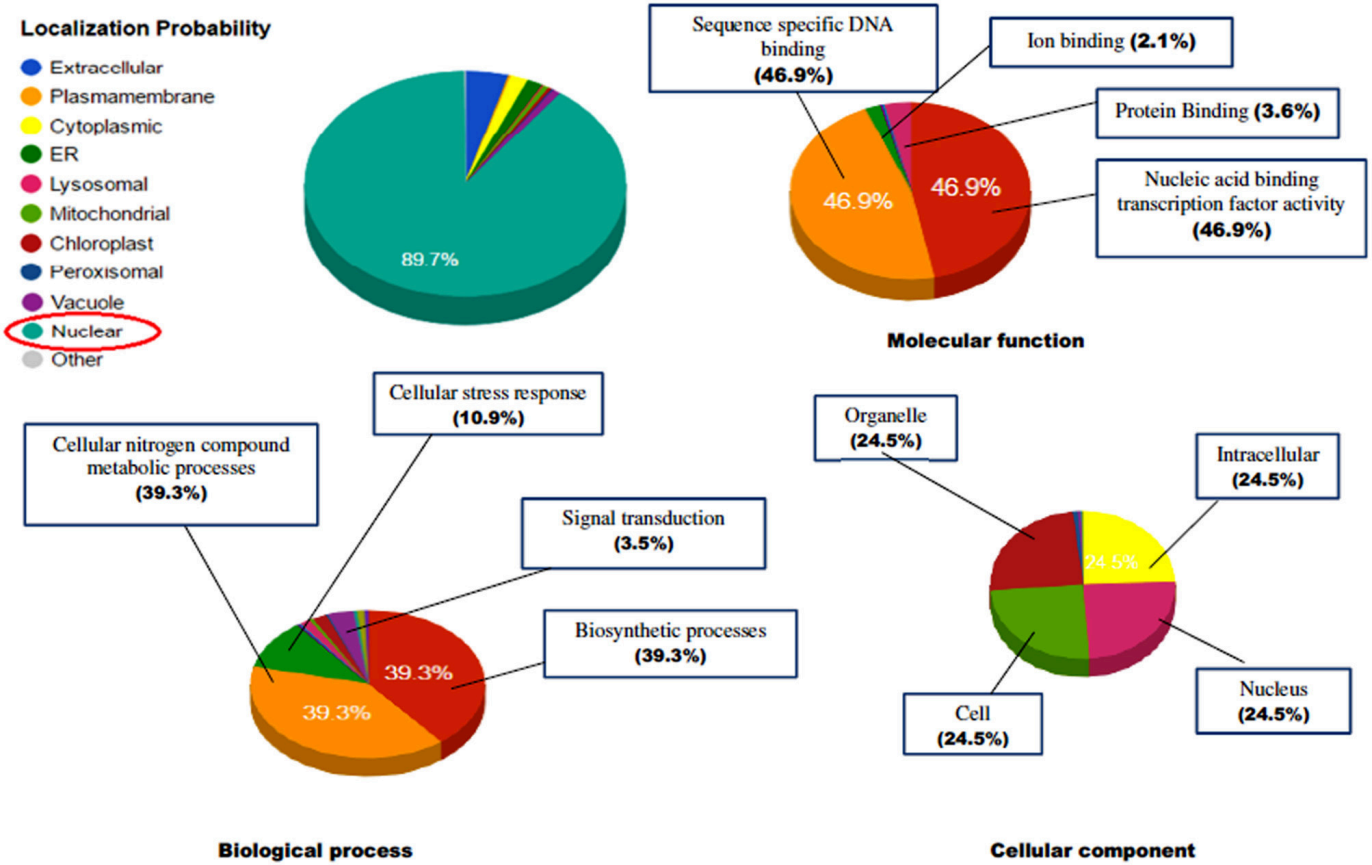
**The prediction of functional gene annotation along with subcellular localization using CELLO2GO web server**. The functional vocabolaries are represented in pie chart digramme evaluating the significant terms in form of their percentage contribution.

The identified protein sequences were submitted to CATH-Gene3D database for their structural classification, functional annotation and characterization as predicted on the behalf of their controlled vocabularies like Gene ontology (GO; Table S3). The structural classification employs the hierarchical clustering of domain structures into evolutionary families and structural groupings, based on sequence and structural similarity. At the lowest levels in the hierarchy, proteins are grouped into evolutionary families (homologous families), for having either significant sequence similarity (35% identity) or high structural similarity and some sequence similarity (20% identity). In our results, the CATH classified the protein to have beta sheet type secondary structure (C-level) (2), single beta sheet (A level) (2.20), with N terminal domain of Tf-II-b (T-level) (2.20.25) and lastly containing WRKY DNA-binding domain (H-level) (2.20.25.80). GO terms are the descriptions of the gene products and are organized around three ontologies that represent molecular function, sub-cellular compartments biological processes involved (Barnawal et al., 2016), where the molecular function term explain the biochemical activity performed by gene product. Biological process term described the ordered assembly of more than one molecular functions. Cellular component term describe the sub-cellular compartments. The ReviGO analysis of the identified vocabularies summarized the long, unintelligible lists of GO terms through searching a representative subset of the terms following a simple clustering algorithm relied on semantic similarity measures. The non-redundant GO term set in ReviGO were visualized in scatter plot digramme based on the numbers associated with GO categories where higher is better with significant GO terms were shown based on unique color and their functional values (Figure 12). Overall, all these terms signify the three separate aspects associated with the biological identity of the gene product. It has been observed that classifications and clustering of proteins to their respective evolutionary families are highly dependent on their sequence or structural similarity or to some extent sequence/functional similarities. In our results, the five significant terms under the biological processes were DNA dependent transcription (GO:0006351), signal transduction (GO:0007165), defense response (GO:0006952), response to chitin (GO:0010200) respiratory burst involved in defense response (GO:0002679). In contrast, the significant terms under the molecular function were sequence specific DNA binding (GO:0043565), sequence-specific DNA binding transcription factor activity (GO:0003700), ADP binding (GO:0043531), and ATP binding (GO:0005524). The cellular location predicted the most possible location of the identified transcript in nucleus (GO:0005634), cytoplasm (GO:0005737), and integral component of membrane (GO:0016021). The characterized gene ontologies were further evaluated for their subcellular localization based on their functional annotation which revealed the protein residing in the nucleus in majority (89.7%) and have characterized to be involved in sequence specific DNA binding activity (46.9%), cellular nitrogen compound metabolic processes (39.3%), biosynthetic processes (39.3%), and also involved in managing stress response (10.9%; Figure 13).

## Discussion

The defense signaling during stress response encompasses a network of signaling events involving multiple partners, and the molecular crosstalk with the expression of multiple genes to enhance the defense mechanism several fold. The actual regulatory mechanism following the stress conditions involves the upregulation of stress responsive genes with the simultaneous activation of some repressors that mediate the gene silencing of other components in the same pathway. Transcription factors (TFs) play crucial roles in mediating the whole process by regulating the genes that may be associated with pathogen-associated molecular pattern-triggered immunity, effector-triggered immunity, hormone signaling pathways, and phytoalexin synthesis (Seo and Choi, 2015). When a plant is subjected to biotic or abiotic stress, the quantitative expression of the WRKY genes is enhanced up to several folds to induce the defense responses by a series of signaling cascades involving endogenous signaling hormones, which protects the plants from abiotic stress challenges or biotic stress damages. The transcriptomic studies following the stress conditions have revealed the upregulation of many defense related WRKY transcription factors (Seo and Choi, 2015). The tomato WRKY genes have been reported to have distinct temporal and spatial expression patterns in different developmental processes and in response to various biotic and abiotic stresses (Huang et al., 2016). Many studies done on gene expression analysis following the exposure of biotic stresses in different species have demonstrated the role of WRKY TFs in defense response against pathogenic challenge. The gene expression analysis following the inoculation with *Botrytis cinerea* through microarray revealed the differential expression of WRKY TFs within 18 h of pathogen infection (Windram et al., 2012). In one study, Liu et al. (2016) reported the increased expression of *WRKY6* gene in the *Solanum pimpinellifolium* cultivar L3708 after the plants found infection with *Phytophthora infestans* and *Botrytis cinerea* or get exposed to other abiotic stress. Similarly, the increased resistance against downy mildew pathogen was demonstrated in five transgenic broccoli lines over-expressing BoWRKY6 (Jiang et al., 2016). The expression of WRKY3 could also be induced by giving wound treatment, or exposure to saline, drought, and cold stress conditions, indicating the pivotal role of the WRKY3 transcription factor in the defense response and other developmental processes. However, the expression of one or more WRKY genes results in a multitude response with different functional aspects and enhances the defense activities several fold. In our previous work done on tomato microarray data sets we have demonstrated the role of SlWRKY4, SlWRKY33 and SlWRKY37 TFs in defense programming against vascular wilt pathogen *Fusarium oxysporum* f. sp. *lycopersici*. The tomato homologs of the *Arabidopsis* WRKY transcription factors 2, 3, 4, 6, 7, 23, 51, 53, and 71 were found to be differentially expressed following the attack of foliar fungal pathogen *Cladosporium fulvum* (van Esse et al., 2009). Moreover, the defense signaling against tomato leaf curl virus involves some WRKY transcription factors including SlWRKY41, SlWRKY42, SlWRKY43, SlWRKY53, SlWRKY54, SlWRKY80, SlWRKY81 (Huang et al., 2016). The tomato WRKY genes have been reported to have distinct temporal and spatial expression patterns in different developmental processes and in response to various biotic and abiotic stresses (Huang et al., 2016).

WRKY TFs have been shown to have preferential binding or interaction with W-box (with core motif TTGACC/T) and clustered W-boxes located in the promoter region of the downstream genes and regulate the dynamic signaling network through kinase or other phosphorylation cascades (Phukan et al., 2016). The motifs and domains located outside the WRKY domain provides binding specificity to WRKYs under different conditions (Phukan et al., 2016). The differences in DNA binding specificities in three group of *Arabidopsis* WRKYs and suggested that other components are essentially required besides the W-box-specific binding to DNA to facilitate a stimulus-specific WRKY function (Brand et al., 2013). It was observed that the DNA binding selectivity of different WRKY members in *Arabidopsis* toward the variants of the W-box embedded in the adjacent DNA sequences, and and determined by additional adjacent DNA sequences lying outside the core TTGACY motif (Ciolkowski et al., 2008). In the conserved WRKYGQK motif the highly conserved glutamine within the β_2_ strand favors the DNA nucleotide bases due to its partial negative charge, whereas the lysine favors to contact with negative charged DNA phosphate backbone (Yamasaki et al., 2012). The mutation experiments have revealed the importance of each conserved residues in DNA-protein interaction as the replacement of each of the conserved residues: Trp, Arg, two Lys, Tyr, and Gly to Ala significantly decreased or almost completely abolished the DNA-binding activity (Grzechowiak, 2014), which also demonstrated the relevance of these amino acid residues in the stabilization of the correct structure of DNA-protein complex and therefore, critical for maintaining DNA-protein interactions (Maeo et al., 2001; Duan et al., 2007). However, W-box non-specific binding were also reported in some species. In *Oryza sativa* WRKY13 (OsWRKY13) binds to both PRE4 element (TGCGCTT) and W-box (Cai et al., 2008). The strong conservation of the residues around the WRKY domain reflects he evidence that least disturbances might have occurred in these regulatory regions in closely related and even more divergent species. These conserved amino acid residues have evolutionary significance as their interaction with specific ligand molecules may trigger response to environmental conditions, and therefore play critical role in providing disease resistance, drought tolerance (Karkute et al., 2015). This evolutionary conservation also explains that with the exception of species specific binding sites, one can expect functional binding sites to be retained among related species. In our results, we have found two WRKY binding domains in each of SlWRKY3 and SlWRKY4 and therefore, classified both SlWRKY3 and SlWRKY4 in group (I) (Rushton et al., 2010). The two WRKY domains in group I proteins play different roles in DNA-binding activities, where the C-terminal domain that plays a major role in binding to the W-box, while the N-terminal WRKY domain increases the binding affinity and provides specificity to bind the target gene (Maeo et al., 2001; Wen et al., 2014). The preferential binding of a group II WRKY transcription factor from *Jatropha curcas*, an important biofuel crop showing 46% identity with *S. tubersosum* to W-box of pathogenesis related-1 (PR-1) and iso1 (encoding isoamylase1) promoters has been well-demonstrated through electrophoretic mobility shift assay (EMSA) results (Agarwal et al., 2014). In recent years, different gene families in plants have been identified and characterized computationally across the genome based on phylogeny, motif composition analysis and their expression profiles. The genome wide analysis of Musa WRKY gene family revealed that during the course of evolution subtle changes in nucleotide sequences resulted into origin of additional or new motifs in two species of banana (*Musa acuminata* and *Musa balbisiana*) which get involved in neo-functionalization of different WRKY members (Goel et al., 2016). Recently, *in silico* genome wide functional characterization of WRKY gene family has been reported in many species including *Salix arbutifolia* (Rao et al., 2015), pepper (*Capsicum annuum* L.) (Cheng et al., 2016). More recently, Zheng et al. (2017) provided an *in silico* genome wide identification, phylogenetic studies and expression analysis of the R2R3-MYB gene family in *Medicago truncatula*. The phylogenetic studies for the origin and evolution of WRKY gene family in rice, tomato and *Arabidopsis* concluded that similar motif composition is shared by tomato WRKYs in each group (Huang et al., 2016).

The protein functional associative interactive network predict the proteins that involve in WRKY signaling cascades and identification and characterization of these interactions are crucial for elucidating the molecular mechanism of signal transduction and metabolic pathways at both the cellular and systemic levels. Due to its large consistent and reliable expression datasets *A. thaliana* coregulatory network can be used as reference for other species, where a smaller set of expression experiments is available (Berri et al., 2009). In addition, for the species having orthologous relationship with *Arabidopsis* the approach can be employed for identifying the existence of genes involved in a common biological process to reveal the existence of co-regulatory networks (Pandey and Somssich, 2009). Berri et al. (2009) demonstrated the WRKY co-regulatory network in *Arabidopsis* and *O. sativa* for 20 pairs of orthologous genes and found that of these 20 gene pairs 8 pairs of genes were coregulated in both species and the results were further confirmed using microarray, quantitative PCR and the results of principle component analysis (PCA).

The experiments of comparative genetic mapping have well-demonstrated the colinearity in the chromosome segments and gene repertoire for more closely related species (Rossberg et al., 2001). In this context, Ku et al. (2000) demonstrated the syntenic conservation of segments in between the genome of tomato and *Arabidopsis*. The BAC clone for chromosome 2 region in tomato showed the conservation of gene content and order with four different segments of *Arabidopsis* chromosomes 2–5. The degree of microcolinerity observed between tomato and *Arabidopsis* could be exploited for localizing orthologous genes in these two separate members in an unambiguous manner (Rossberg et al., 2001). Molan and El-Komy (2010) demonstrated that some WRKY genes of *S. lycopersicum* were found to be phylogenetically closer to WRKY genes of *A. thaliana, S tubersosum, O. sativa*, and *N. tabacum*. We have generated the the Circos visualization maps (Krzywinski et al., 2009; Figure 7) using the WRKY3 and WRKY4 protein sequences from all the respective members of tomato family and compared their evolutionary relationship with the model *A. thaliana* to facilitate the identification and analysis of similarities and differences that get arised from genome comparisions. Similarly, sequence alignment between AtWRKY33 and the two tomato WRKY33 homologs showed extensive sequence similarity over the entire proteins including the extended CTDs (Zhou et al., 2014). Therefore, the principle of comparative genomics allowed for the comparative analysis of entire gene regulatory networks across all the eukaryotes (Thompson et al., 2015). Recently, Yue et al. (2016) developed a Predicted Tomato Interactome Resource (PTIR), based on experimentally determined orthologous interactions in six model organisms including *Arabidopsis*, nematode worm, fruit fly, human, rice, and yeast, reported that, *Arabidopsis* shares the highest evolutionary conservation with tomatoes. PTIR database represents a centralized platform to integrate the information pertaining to protein-protein interaction, functional annotation, ortholog mapping, and domain architecture in the tomato proteome and their reliability is based on shared GO terms, co-expression, co-localization as well as available domain-domain interactions. These established interactomes could be served as repositories to predict PPIs of other species on a genome-wide scale (Yue et al., 2016). This interlogs base protein-protein interaction approach has provided the most possible and probable interactive partners involved in these interactions. In another study, Yang et al. (2013) inferred the *Brassica rapa* interactome using protein-protein interaction data from *A. thaliana*. In our results, we have shown the possible interactive partners for SlWRKY3 that get involved in protein associative interaction network as revealed through STRING server. However, the functional annotation through gene enrichment analysis predicts the functional dimension of the characterized WRKYs based on their controlled vocabularies and specified with GO identities. These interactive associative networks are derived from high throughput experimental data, from the mining of databases and literature, and from predictions based on genomic context analysis (Von Mering et al., 2005). The major interacting partner for tomato WRKY3 was reported to be TRANSPARENT TESTA GLABRA 1 protein, homeobox leucine zipper protein GLABRA2 like and GLABRA3 like transcription factors. The role of WRKY transcription factors in developmental processes is although not much reported however, TRANSPARENT TESTA GLABRA2 (TTG2) provides an exception and play a crucial role in trichome development and also effects mucilage and tannin synthesis in the seed coat (Johnson et al., 2002). The molecular mechanism for regulating the expression of TTG2 involves bHLH (basic helix-loop-helix) and R2R3 MYB transcription factors such as WEREWOLF, GLABRA1 and TRANSPARENT TESTA, and further the TTG2 regulates the expression of GLABRA2. In double mutant studies Johnson et al. (2002) reported that both GL1 and TTG1 are required for the proper functioning of another protein TRANSPARENT TESTA GLABRA 2 (TTG2) which shares function with GLABRA2 in controlling trichome outgrowth in the trichomes of the leaf surfaces. Moreover, the TTG2 proteins show some structural homology around a domain found in WRKY members and also has a two highly conserved sequence motifs, the WRKYGQK amino acid sequence near the N-terminal region (Rushton et al., 1996) and a conserved C-X4-5-C-X22-23-H-X1-H sequence that resembles zinc finger motifs (de Pater et al., 1996; Rushton et al., 1996) which have demonstrated that TTG2 protein act as WRKY like transcription factor (Ishida et al., 2007). Furthermore, it has been reported that in *Arabidopsis* the bHLH transcription factors [GLABRA3 (GL3) and ENHANCER OF GLABRA 3 (EGL3)] are central regulators of trichome and root-hair development, and the same homologous genes in tomato (SlTRY and SlGL3) were identified in tomato, and their transformation in *Arabidopsis* inhibited trichome formation and enhanced root-hair differentiation by strongly repressing GL2 expression. Moreover, the GL3:SlGL3 transformation did not show any obvious effect on trichome or non-hair cell differentiation (Tominaga-Wada et al., 2013). Since the phylogenetic analysis revealed a close relationship between the tomato and *Arabidopsis* genes (Tominaga-Wada et al., 2013). The tomato and *Arabidopsis* partially use similar proteins for regulating the epidermal cell differentiation, trichome initiation, root hair differentiation, and anthocyanin accumulation (Tominaga-Wada et al., 2013; Wada et al., 2014, 2015). These protein-protein interaction studies revealed the possible interactions observed between WRKYs and other proteins as it was demonstrated that transient expression analysis revealed that the activation of GLABRA2 may require concurrent binding of GLABRA1 and GLABRA3 to the Promoter of GLABRA2 (Wang and Chen, 2008). In our results, we have found that tomato WRKY3 and the tomato homologs of *Arabidopsis* showed various direct and indirect interactive partners from high to medium or medium to low confidence levels. However, at very high confidence intervals only one interacting partners SUMO proteins were found and the same was submitted to STRING server. Today, the most effective method for evaluating PPIs at genome wide scale is yeast two-hybrid (Y2H) screening, although this method shows a high rate of false-positives (Kim et al., 2016). The WRKYs- SUMO interaction has been experimentally determined by gold standard protein interaction techniques such as Affinity Capture-MS assay (Miller et al., 2010). In contrast, the indirect protein intraction partners or those that were found to be part of interactome and achieved at medium confidence level such as GLABRA3, GLABRA1, and TRANSPARENT TESTA GLABRA1 (TTG1) were experimentally demonstrated through yeast two hybrid assay to be involved in physical interaction with each other (Payne et al., 2000). Similarly, Balkunde et al. (2011) demonstrated through yeast two hybrid interaction assay the direct interaction observed between TTG1 and GL3 whose homolog proteins in tomato was reported SlGL3 as GLABRA3 (GL3) traps the trichome-promoting factor TRANSPARENT TESTA GLABRA1 (TTG1), the WD repeat protein, in trichomes that, in turn, results in a depletion of TTG1 in trichome neighboring cells (Balkunde et al., 2011). Recently, the protein-protein interaction between GL1, GL3, or GL3 TTG1 have been experimentally demonstrated through yeast three hybrid assays, pulldown experiments (luminescence-based mammalian interactome), and fluorescence lifetime imaging microscopy-fluorescence resonance energy transfer studies (Pesch et al., 2015) Similarly, Zhang et al. (2003) through yeast two hybrid assay and plant overexpression studies demonstrated that that ENHANCER OF GLABRA3 (EGL3) like GL3 interact with TTG1, the myb proteins GL1, PAP1 and PAP2, CPC and TRY (in tomato homologs SlTRY) and it will form heterodimers with GL3. The immunity to the plant is well-achieved by two structurally similar, but distinct classes of WDR-containing proteins that includes Gβ and TRANSPARENT TESTA GLABRA1 (TTG1). These two proteins provides two independent ternary protein complexes that function at opposite ends of a plant immune signaling pathway (Miller et al., 2016).

The role WRKY3 and WRKY4 has been investigated to understand their expression in case of necrotrophic as well as biotrophic pathogens. This role has been confirmed by a comparative study in which when either one or both of the *WRKY3* and *WRKY4* genes were mutated, the single or double mutants thus obtained had exhibited higher susceptibility to fungal pathogens and supported higher fungal growth (Lai et al., 2008). The transgenic overexpression line for *AtWRKY3* and *AtWRKY4* generated through T-DNA insertion mutants did not have major effect on plant response to pathogens such as *Pseudomonas syringae* however, the over expression of *AtWRKY4* alone resulted into higher susceptibility to the bacterial pathogen by suppressing the pathogen-induced PR1 gene expression. These studies strongly support that WRKY3 and WRKY4 proteins play their crucial role in regulating the plant defense against necrotrophic pathogens but have negative role in tackling the biotrophic pathogens. Moreover, the extensive cross communication that occur between these two hormone signaling pathways regulates fine tuning of defense related transcriptional programming, which determines resistance to the invaders and trade-offs with plant development. The regulatory switches for the expression of these two signaling cascades are directly or indirectly correlated with multiple and diverse processes along with the involvement of other signal molecules.

## Conclusions

Structural and functional elucidation of WRKY transcriptional factors through computational approaches provides a direct insight into the sequence specific features associated with functional redundancy found within members that regulates the stimulus bound transcriptional reprogramming of stress responsive genes. Since, the protein structure is more conserved than sequence and the structure-function relationship is even more complex than the relationship between sequence and structure. The WRKY gene expression analysis following the exposure of different abiotic and biotic stresses when compiled with the data as obtained through GO annotations may reveal the functional dimension of individual WRKY proteins, and thus would be helpful in their functional characterization in a stimulus dependent manner. Furthermore, both the structural and functional characterization of WRKY proteins would provide necessary information regarding their phylogenetic relationships, ancestral origins, divergence and other evolutionary parameters for comprehensive study of function-adaptive process, thus the regulatory mechanisms of WRKY superfamily genes in tomato and other related crops. This approach will be helpful in developing transgenics with improved agronomic traits and have potential to counteract both abiotic and biotic stresses. In this work, we have demonstrated the interaction of W-box DNA with the prominent residues of WRKY domain that makes this interaction more feasible and favorable, and assist in the fine-tuning of gene regulation. Moreover, the interacted residues of SlWRKY3 and SlWRKY4 showing similarity with reported DNA-protein complex of AtWRKY4 and this investigation confirms that the identified genes *SlWRKY3* and *SlWRKY4* may show possible role in mitigating abiotic stresses apart from contributing defense signaling against plant diseases.

## Author contributions

MA designed and planned the experiment. Did all the experiments and analyzed the data generated and finally prepared and wrote the manuscript. VS assisted in some experimental sections and did the computational analysis of the results. MM assisted in verifying and editing the final version of the manuscript and prepared the manuscript as required by the journal guidelines. VG assisted in manuscript editing, data evaluation. RU helped in reformulating the manuscript and drafted the manuscript more informative. SS helped in the manuscript writing data evaluation, analysis of results, and resolving critical questions related to the manuscript.

### Conflict of interest statement

The authors declare that the research was conducted in the absence of any commercial or financial relationships that could be construed as a potential conflict of interest.
