# Supplementary material for: Structural and Functional Insights into WRKY3 and WRKY4 Transcription Factors to Unravel the WRKY–DNA (W-Box) Complex Interaction in Tomato (Solanum lycopersicum L.). A Computational Approach
Source: Front Plant Sci. 2017 May 29;8:819. doi: 10.3389/fpls.2017.00819 (PMC5447077; doi:10.3389/fpls.2017.00819)
Supplement: Supplementary file 4 [file DataSheet1.DOCX]

**All Supplementary Information with Figure Legends and Tables**

**Figure S1.** Multiple sequence alignment for WRKY3 protein from all the respective members of tomato family showing strong conservation of residues as revealed through CLC bio workbench.

(Figure S1 given in separate PDF Image)

**Figure S2.** Multiple sequence alignment for WRKY4 protein from all the respective members of tomato family showing strong conservation of residues as revealed through CLC bio workbench.

(Figure S2 given in separate PDF Image)


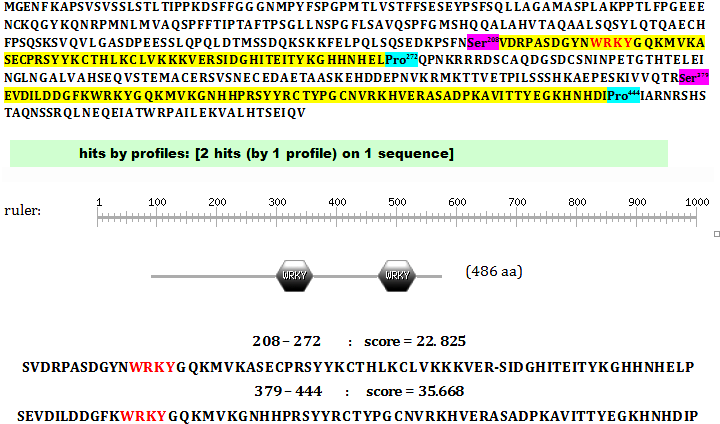


**Figure S3.** The presence of two WRKY domains in SlWRKY3 as revealed by ExPASy-PROSITE tool. The functional signature sequences at both the N-terminal and C-terminal end have been highlighted.


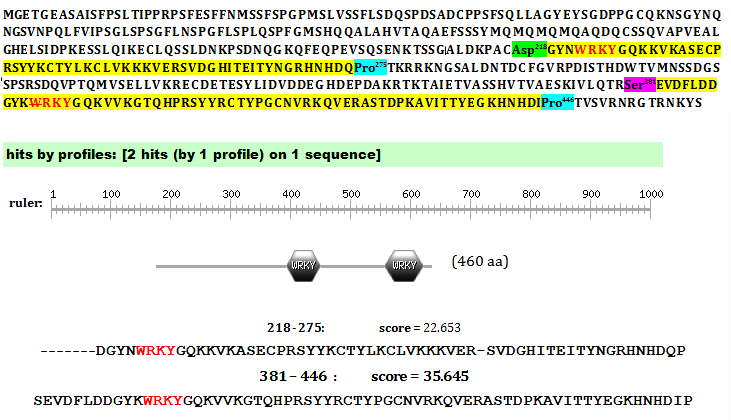


**Figure S4.** The presence of two WRKY domains in SlWRKY4 as revealed by ExPASy-PROSITE tool. The functional signature sequences at both the N-terminal and C-terminal end have been highlighted.


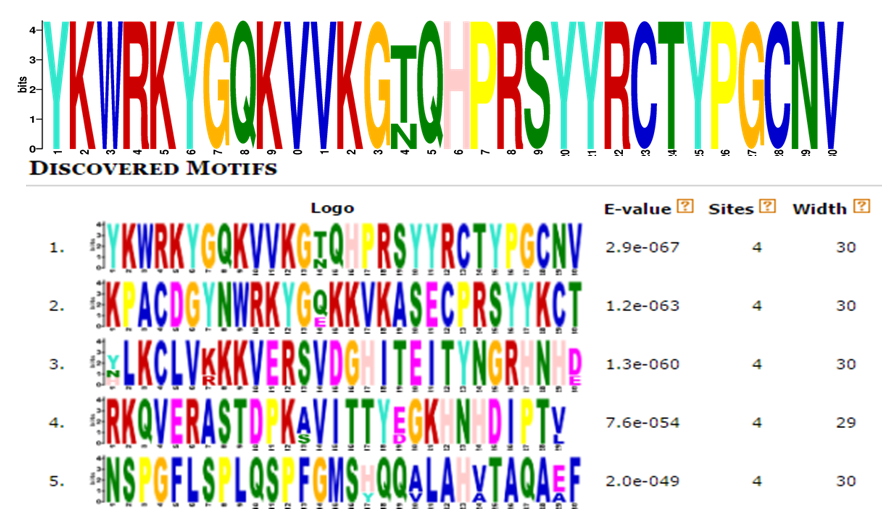


B

A

**Figure S5.** Motif scan using MEME analysis for WRKY3 protein. **(A)** The first five motifs in WRKY3 protein as discovered using MEME and MAST. The E-value given here predicts the statistical significance of each motif it finds and is a fairly conservative estimate of the likewise occurrence value of that motif. **(B)** The logo of the motif 2 showing consensus WRKY sequences found in all the members.


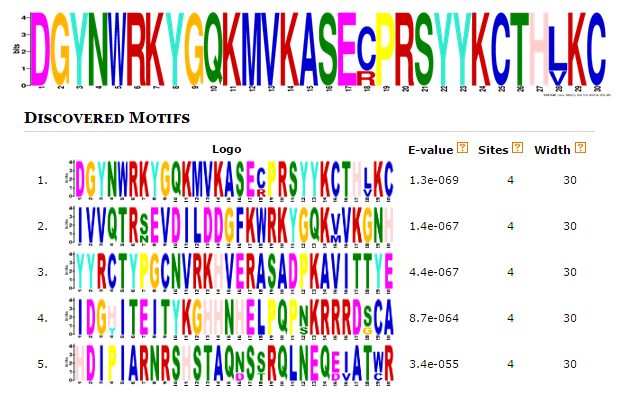


B

A

**Figure S6.** Motif scan using MEME analysis for WRKY4 protein. **(A)** The first five motifs in WRKY4 protein as discovered using MEME and MAST.The E-value given here predicts the statistical significance of each motif it finds and is a fairly conservative estimate of the likewise occurrence value of that motif. **(B)** The logo of the discovered motif1 that constitutes the C-terminal WRKY domain and have consensus WRKY sequence and present in all the respective members of tomato family.


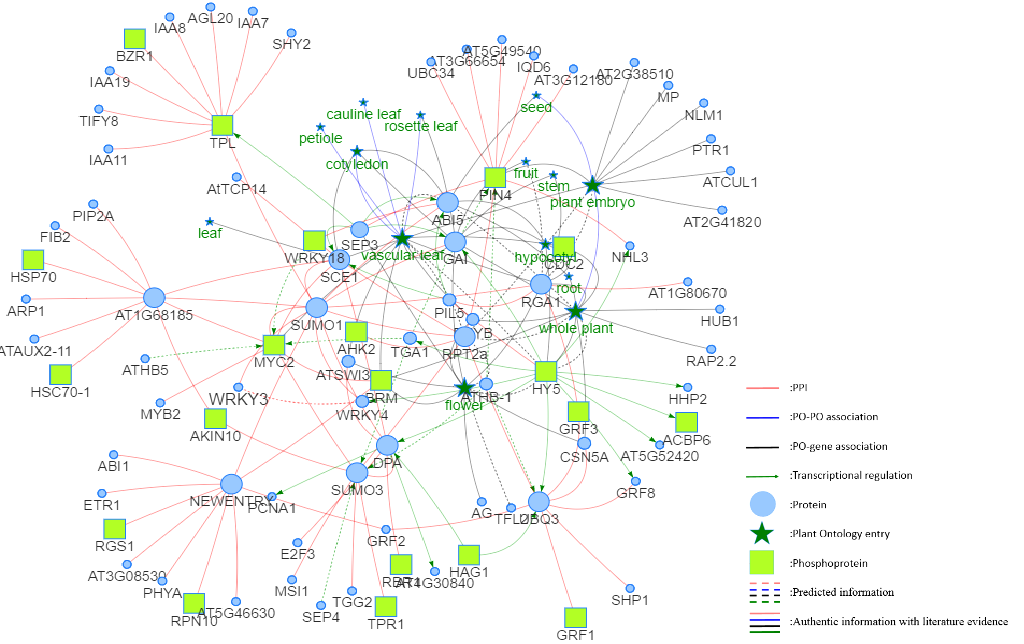


**Figure S7.** The interactome network for *Arabidopsis* WRKY3 and WRKY4 proteins as revealed through Predicted Tomato Interactome Resources (PTIR) showing the direct and indirect (dotted red line) possible interactive partners and obtained through shared GO terms, co-expression, co-localization as well as available domain-domain interactions. The sky blue circles represent proteins that get participate in interaction and the green square represent the potential phosphorylated proteins.


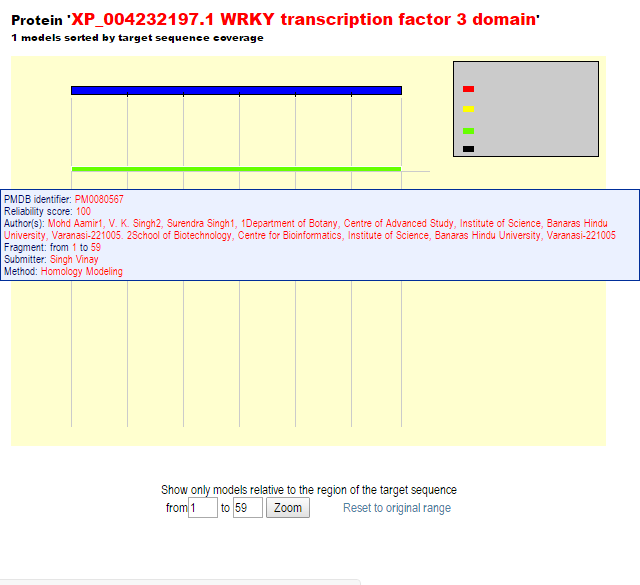


B

A


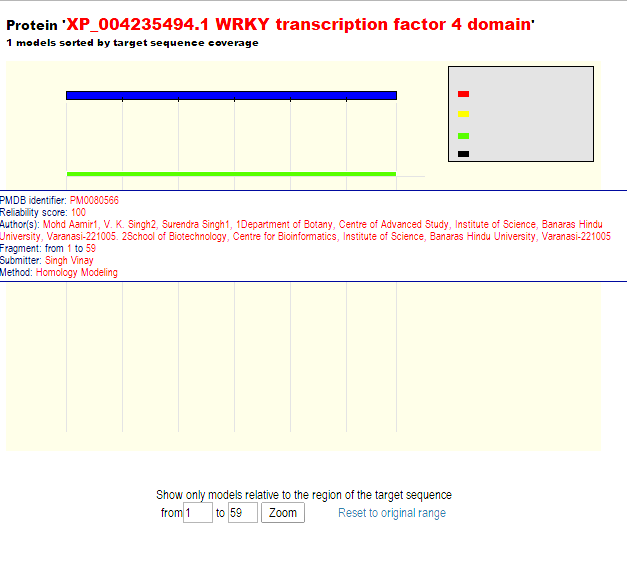


**Figure S8. (A)** Acknowledgement details of the submitted protein models of SlWRKY3 at PMDB database with their PMDB IDs author details, methods employed and reliability score values. **(B)** Details of the submitted protein models of SlWRKY4.


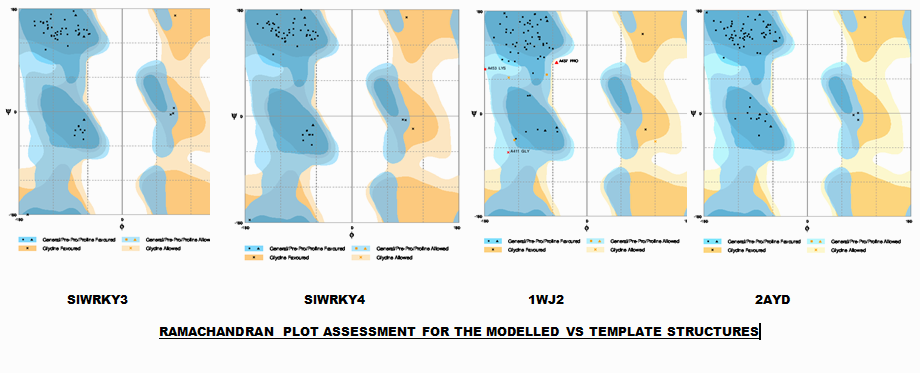


**Figure S9.** Ramachandran plot statistics as revealed through RAMPAGE server revealing the displays the psi (𝜓) and phi (𝜑) backbone conformational angles for each residue in a protein.

A

BBB


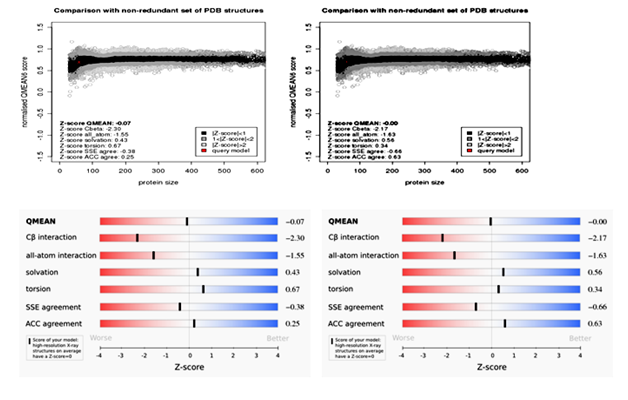


D

C

**Figure S10.** Graphical representation of energy values pertaining to Q MEAN Score. QMEAN Z-score is a measure for absolute quality of the model. QMEAN score of the model was compared to the score of high-resolution reference structure solved by X-ray crystallography. All the statistical Z-score terms were depicted in left corner of the figure and reliable range of Z-score for model quality was also given in right corner. Red color legend indicates the Z-score QMEAN that is, observed as -0.07 in case of WRKY3 **(A)** and -0.00 for WRKY4 **(B)** Different score values determining the qualitative aspects of modeled SlWRKY3 **(C)** and SlWRKY4 **(D)**


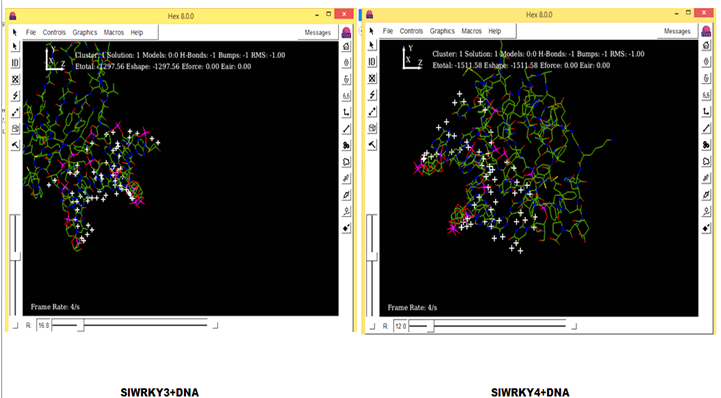


**Figure S11.** DNA-protein docking studies using Hex server. The minimum interaction energies for the most stable docked complexes have been shown on windows screen. The negative values of energy score indicate the most possible and stable orientation of the ligand molecules with their receptor.

**Table S1.** Functional annotation, accession ID and interacting score values for both first and second shell of interactors that form mutual interactive associative network along with SlWRKY3. The higher value of score indicate the more frequent interaction exist between two associated proteins.

(Data given in separate MS-excel file)

**Table S2.** Functional annotation, accession ID and interacting score values for both first and second shell of interactors that form mutual interactive associative network along with SlWRKY4.

(Data given in separate MS-excel file)

**Table S3.** The raw scores, Z-scores of the QMEAN composite score as well as all terms are provided relating the quality estimates to scores obtained for high-resolution reference structures solved experimentally by X-ray crystallography.

| **WRKY 3** | | | **WRKY 4** | |
| --- | --- | --- | --- | --- |
| **Scoring Function term** | **Raw Score** | **Z Score** | **Raw Score** | **Z Score** |
| C-β interaction energy | 9.57 | -2.3 | 8.08 | -2.17 |
| All-atom pairwise energy | -418.37 | -1.55 | -373 | -1.63 |
| Solvation energy | -4.28 | 0.43 | -5.01 | 0.56 |
| Torsion angle energy | -19.57 | 0.67 | -17.09 | 0.34 |
| Q Mean Score | 0.696 | -0.07 | 0.706 | 0 |

**Table S4.** Gene ontology enrichment analysis summarizing the functional annotation from all the controlled vocabularies including biological process, molecular function, and cellular processes. The redundant GO terms have been displayed in the form of scattered plot values and all the functional annotations with the Gene ontology IDs have been shown in table along with their frequency and other values.

(Data given in separate MS-excel file)
