# Supplementary figures and images for: Structural and Functional Insights into WRKY3 and WRKY4 Transcription Factors to Unravel the WRKY–DNA (W-Box) Complex Interaction in Tomato (Solanum lycopersicum L.). A Computational Approach
Source: Front Plant Sci. 2017 May 29;8:819. doi: 10.3389/fpls.2017.00819 (PMC5447077; doi:10.3389/fpls.2017.00819)

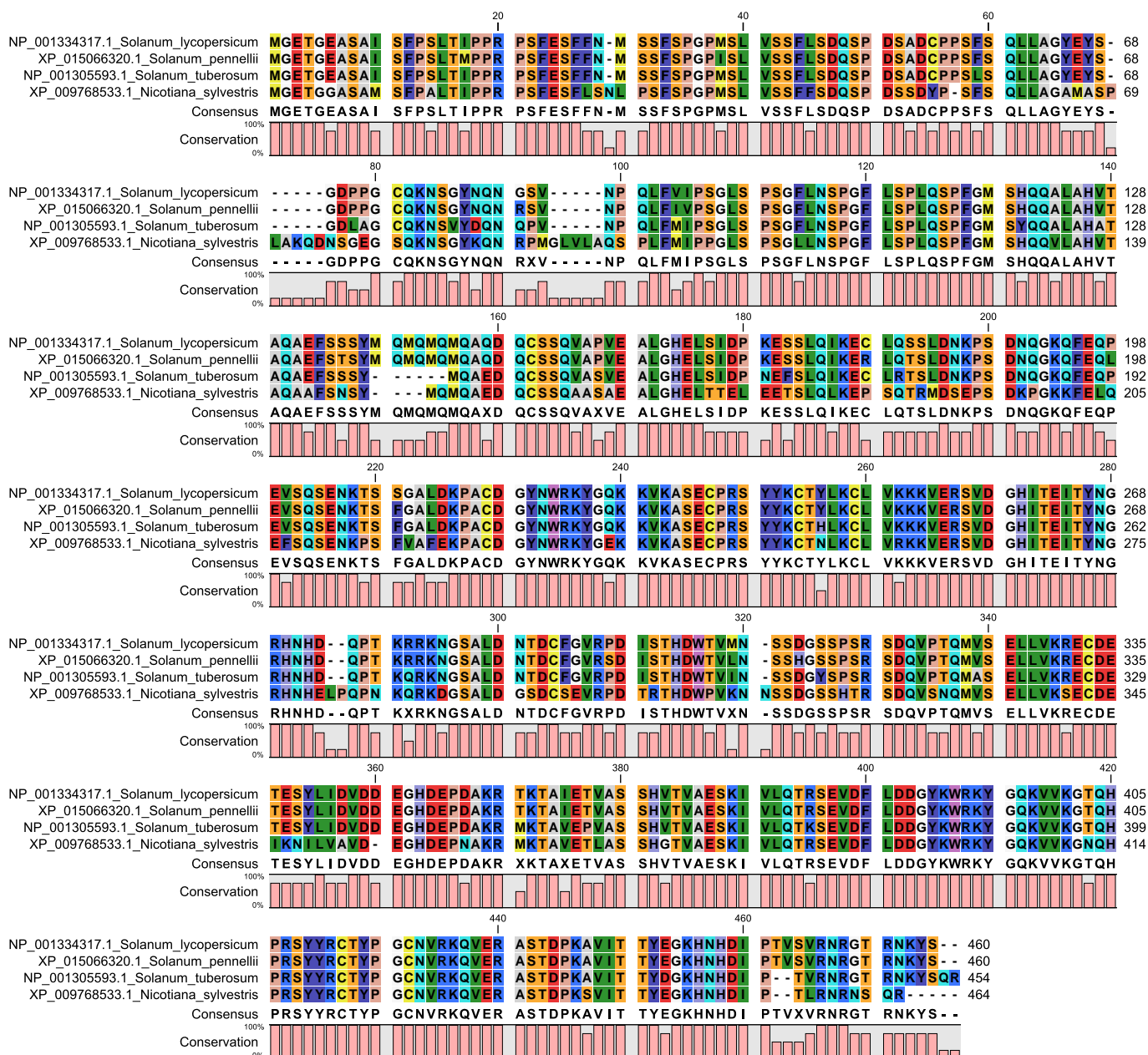

Supplement: Supplementary file 5 [file Image1.PDF]

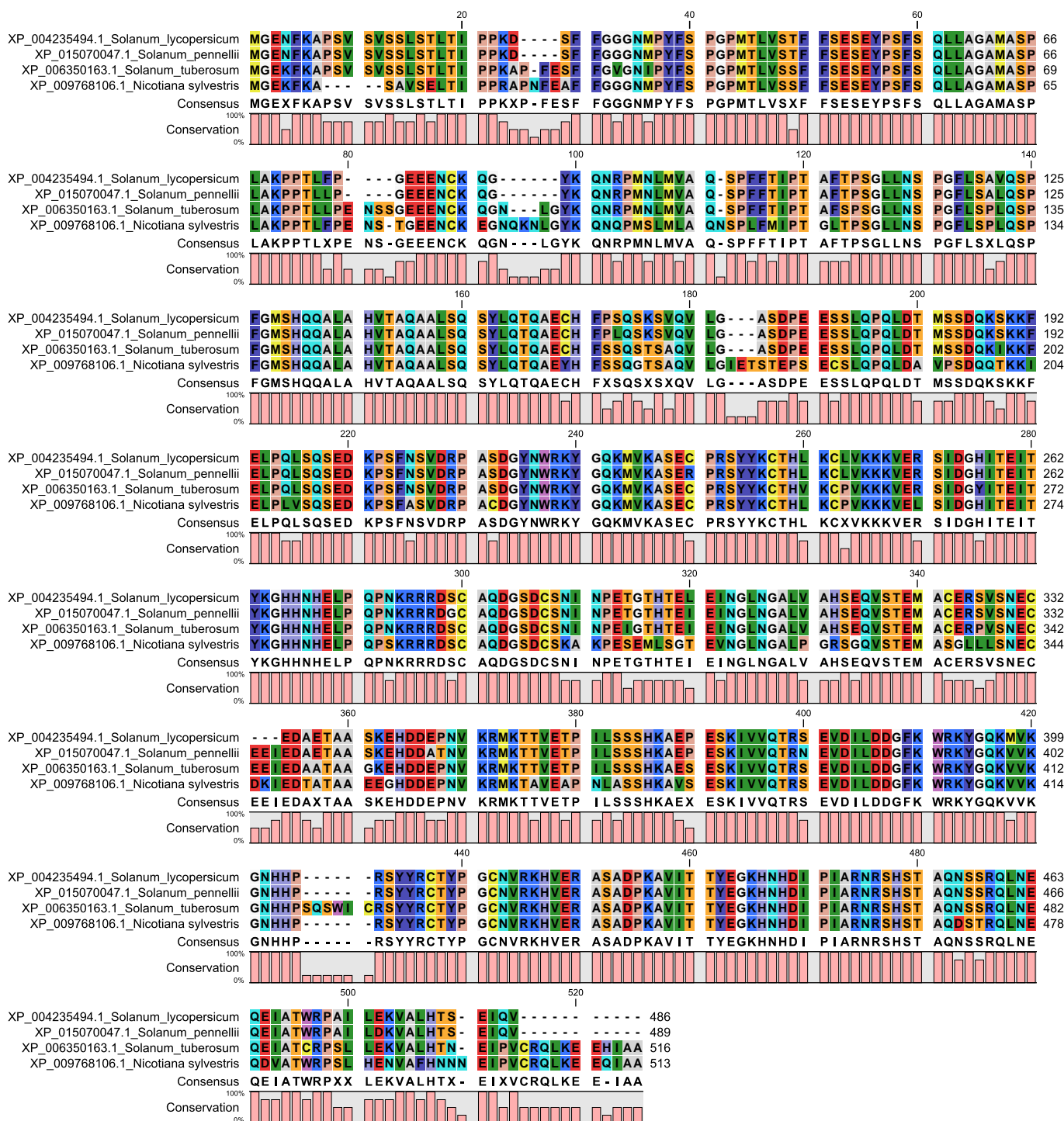

Supplement: Supplementary file 6 [file Image2.PDF]
